# Interactions of hydrolyzed β-lactams with the L1 metallo-β-lactamase: Crystallography supports stereoselective binding of cephem/carbapenem products

**DOI:** 10.1016/j.jbc.2023.104606

**Published:** 2023-03-15

**Authors:** Philip Hinchliffe, Karina Calvopiña, Patrick Rabe, Maria F. Mojica, Christopher J. Schofield, Gary I. Dmitrienko, Robert A. Bonomo, Alejandro J. Vila, James Spencer

**Affiliations:** 1 School of Cellular and Molecular Medicine, University of Bristol, Biomedical Sciences Building, University Walk, Bristol, United Kingdom; 2 Chemistry Research Laboratory, Department of Chemistry and the Ineos Oxford Institute for Antimicrobial Research, University of Oxford, Oxford, United Kingdom; 3 Department of Molecular Biology and Microbiology, School of Medicine, Case Western Reserve University, Cleveland, Ohio, USA; 4 U.S. Department of Veterans Affairs, CWRU-Cleveland VA Medical Center for Antimicrobial Resistance and Epidemiology (Case VA CARES), Cleveland, Ohio, USA; 5 Research Service, Louis Stokes Cleveland Department of Veterans Affairs Medical Center, Cleveland, Ohio, USA; 6 Grupo de Resistencia Antimicrobiana y Epidemiología Hospitalaria, Universidad El Bosque, Bogotá, Colombia; 7 Department of Chemistry, University of Waterloo, Waterloo, Ontario, Canada; 8 School of Pharmacy, University of Waterloo, Waterloo, Ontario, Canada; 9 Departments of Medicine, Biochemistry, Pharmacology, and Proteomics and Bioinformatics, Case Western Reserve University School of Medicine, Cleveland, Ohio, USA; 10 Laboratorio de Metaloproteínas, Instituto de Biología Molecular y Celular de Rosario (IBR, CONICET-UNR), Rosario, Argentina; 11 Área Biofísica, Facultad de Ciencias Bioquímicas y Farmacéuticas, Universidad Nacional de Rosario, Rosario, Argentina

**Keywords:** metallo β-lactamase, antibiotic resistance, β-lactam, carbapenem, hydrolysis, X-ray crystallography, L1 carbapenemase

## Abstract

L1 is a dizinc subclass B3 metallo-β-lactamase (MBL) that hydrolyzes most β-lactam antibiotics and is a key resistance determinant in the Gram-negative pathogen *Stenotrophomonas maltophilia*, an important cause of nosocomial infections in immunocompromised patients. L1 is not usefully inhibited by MBL inhibitors in clinical trials, underlying the need for further studies on L1 structure and mechanism. We describe kinetic studies and crystal structures of L1 in complex with hydrolyzed β-lactams from the penam (mecillinam), cephem (cefoxitin/cefmetazole), and carbapenem (tebipenem, doripenem, and panipenem) classes. Despite differences in their structures, all the β-lactam-derived products hydrogen bond to Tyr33, Ser221, and Ser225 and are stabilized by interactions with a conserved hydrophobic pocket. The carbapenem products were modeled as Δ^1^-imines, with (2*S*)-stereochemistry. Their binding mode is determined by the presence of a 1β-methyl substituent: the Zn-bridging hydroxide either interacts with the C-6 hydroxyethyl group (1β-hydrogen-containing carbapenems) or is displaced by the C-6 carboxylate (1β-methyl-containing carbapenems). Unexpectedly, the mecillinam product is a rearranged N-formyl amide rather than penicilloic acid, with the N-formyl oxygen interacting with the Zn-bridging hydroxide. NMR studies imply mecillinam rearrangement can occur nonenzymatically in solution. Cephem-derived imine products are bound with (3*R*)-stereochemistry and retain their 3′ leaving groups, likely representing stable endpoints, rather than intermediates, in MBL-catalyzed hydrolysis. Our structures show preferential complex formation by carbapenem- and cephem-derived species protonated on the equivalent (β) faces and so identify interactions that stabilize diverse hydrolyzed antibiotics. These results may be exploited in developing antibiotics, and β-lactamase inhibitors, that form long-lasting complexes with dizinc MBLs.

Antimicrobial resistance (AMR) is a growing health and economic threat and has been estimated to be directly responsible for 1.27 million deaths worldwide in 2019 (1). β-Lactams are the most prescribed and the most clinically important antibiotics used to treat bacterial infections (2, 3). Extensive development of bicyclic β-lactams over decades has led to various classes that differ in the nature of the ring that is fused to the four-membered β-lactam ring and of the substituent groups, in particular at the C-6/C-7 and C-2/C-3 positions (Fig. 1*A*) (4, 5).

**Figure 1:**
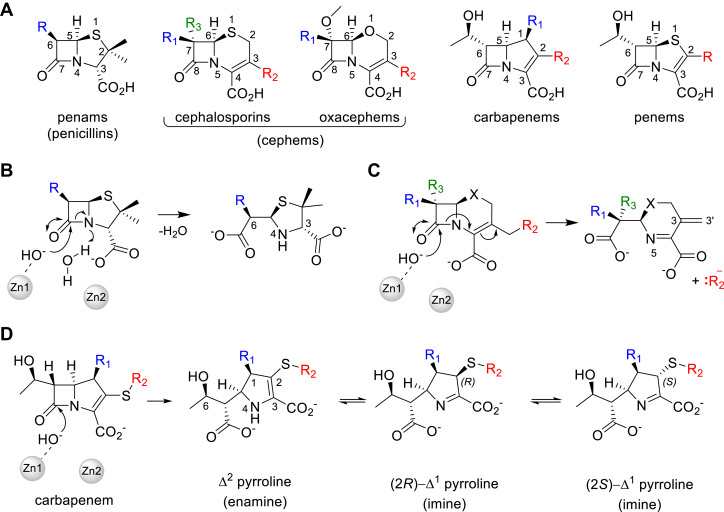
**Bicyclic β-lactams and their major hydrolysis products formed on reaction with metallo-β-lactamases.***A*, major classes of bicyclic β-lactam antibiotics, with variable substituent (R)-groups colored. *B*, penam/penicillin degradation by MBLs. In the resting state, a water/hydroxide bridges the dizinc MBL center. This becomes terminal to Zn1 and is activated to attack the β-lactam carbonyl, opening the β-lactam ring, with subsequent protonation of N-4. *C*, the degradation pathway of cephems with a C-3′ leaving group can result in loss of the C-3 R_2_ substituent, without protonation of the β-lactam N-5 nitrogen. *D*, tautomerization of carbapenem-derived Δ^2^ pyrroline hydrolysis products to give (*R*/*S*)-Δ^1^ pyrrolines *via* protonation at C-2.

The penam/penicillin scaffold constitutes a fusion of the β-lactam ring with a five-membered thiazolidine derivatized with a gem-dimethyl group at C-2 and a variable C-6 substituent (R-group in Fig. 1*A*) (5). Cephems contain either 6-membered dihydrothiazines (cephalosporins) or dihydrooxazines (oxacephems) as the fused partner ring (6, 7). They are the most extensive β-lactam class, with dozens of different compounds derivatized at C-3 and C-7 (R_1_–R_3_ groups, Fig. 1*A*) (8, 9). Cephems that contain a C-7 methoxy group (R_2_ in Fig. 1*A*), for example, cefoxitin and cefmetazole, are referred to as 7α-methoxy cephalosporins (10), second-generation cephalosporins or cephamycins (11). Those that have a bulky C-7 substituent (*e.g.*, ceftazidime) containing an oxyimino group are referred to as third-generation- or extended-spectrum oxyimino-cephalosporins and have an extended spectrum of activity compared to earlier cephalosporins (12). Clinically used penems are synthetic compounds that were developed with the aim of combining the properties of cephalosporins and penams, with the β-lactam ring fused to a five-membered thiazoline ring (13). Only one penem, faropenem (which contains a C-2 tetrahydrofuran substituent), is clinically available and is used extensively in Asia to treat respiratory and urinary tract infections (14, 15). The carbapenems contain a five-membered pyrroline ring as the fusion partner and possess the broadest spectrum of activity amongst β-lactams (16). The carbapenem scaffold contains a variable substituent group (R_2_ in Fig. 1) at C-2, a methyl or hydrogen at C-1 (termed the 1β-methyl/hydrogen group) and a small α-hydroxyethyl substituent at C-6 that contrasts with the larger and diverse C-6/C-7 β-substituents of penicillins and cephems. Carbapenems with a 1β-methyl rather than 1β-hydrogen are particularly useful medicines as they are less susceptible to hydrolysis by human dehydropeptidase I (DHP-I) and so do not need to be co-administered with a DHP-I inhibitor such as cilastatin (16, 17).

The efficacy of all β-lactams is increasingly being diminished due to the emergence and reemergence of β-lactamases that hydrolyze the β-lactam ring to render them inactive. β-Lactamase enzymes are grouped into four main classes (18): A, C, and D use a serine side chain hydroxyl group as a nucleophile for catalysis (serine-β-lactamases, SBLs), while the class B enzymes employ a zinc-bound hydroxide ion (metallo-β-lactamases, MBLs) (4). MBLs are further divided, based on their sequences and Zn ion requirements, into the dizinc subclass B1 and B3 enzymes and the monozinc subclass B2 enzymes (19, 20). B1 and B3 MBLs hydrolyze all bicyclic β-lactam classes, while the B2 enzymes are predominantly carbapenemases, but all classes do not hydrolyze the monocyclic monobactams. The active sites of dizinc MBLs such as L1 contain two closely spaced (*c*. 3.6 Å) zinc ions, termed Zn1 and Zn2, bridged by the hydroxide nucleophile, thought, respectively, to activate the β-lactam carbonyl for nucleophilic addition and stabilize accumulating negative charge on the β-lactam amide N.

Catalysis by dizinc MBLs proceeds without the formation of a covalent intermediate and involves an addition to the β-lactam carbonyl (and subsequent opening of the β-lactam ring) by an activated and terminal Zn-coordinated hydroxide ion (4, 21, 22). In penams (Fig. 1*B*) the β-lactam N is then protonated, resulting in the formation of an inactive penicilloic acid. Cephem hydrolysis can result in different products (5), but β-lactamase-mediated hydrolysis of cephalosporins with a C-3′ leaving group (which include most in current clinical use) is usually reported to result in a product that has lost its C-3 substituent with the formation of an exocyclic methylene group (C-3 = C-3′ double bond) (5, 23, 24, 25, 26, 27) (Fig. 1*C*). This mechanism of cephem product generation, involving loss of the 3′ leaving group, has been exploited in the development of fluorogenic reporter substrates for SBLs and MBLs (28) and also for MBL inhibitors (29). Carbapenem breakdown is initiated in a similar fashion to that of penams but is thought to (predominantly) produce a nascent Δ^2^-enamine product that tautomerizes to give two possible Δ^1^-imine species in which C-2 isomerizes between (*R*) and (*S*) configurations (Fig. 1*D*) (4, 5, 22, 30, 31, 32).

L1 is a dizinc subclass B3 MBL that is chromosomally encoded in *Stenotrophomonas maltophilia* (33), a Gram-negative bacterium that is an important cause of healthcare-associated infections (34, 35, 36, 37). *S. maltophilia* is particularly problematic in debilitated and immunocompromised patients, such as those with cystic fibrosis (35) but also increasingly in immunocompetent individuals (38). *S. maltophilia* is therefore recognized by the World Health Organization (WHO) as an organism for which treatment strategies must be developed. Many L1 variants have now been identified in clinical *S. maltophilia* isolates, with sequence identities ranging as low as 76% to the originally characterized L1 from strain IID 1275 (39, 40). These variants have not been extensively characterized *in vitro* but one (referred to as L1e) hydrolyzes nitrocefin and imipenem more poorly than the parent enzyme (39). Herein, we refer to the originally characterized enzyme as L1.

Understanding the interactions that stabilize hydrolyzed antibiotics in the active site of dizinc MBLs is key to developing novel antibiotics that are more poorly hydrolyzed by these enzymes as well as inhibitors that can exploit such interactions to generate long-lasting complexes. Here, we report the use of high-resolution X-ray crystallography to explore the interactions of six different hydrolyzed β-lactams, with the originally described, and most extensively characterized L1 enzyme that is a major contributor to *S. maltophilia* β-lactam resistance. The β-lactams studied are representatives from three chemically distinct classes, carbapenems, penems, and cephems, with varying proposed hydrolysis mechanisms and expand the available crystal structures of L1 with hydrolyzed products to cover all the major bicyclic β-lactam classes. These data identify multiple stable complexes formed between L1 and β-lactam-derived products with different chemical structures, revealing interactions common to these diverse scaffolds. Hydrolyzed carbapenems and cephems are bound in forms protonated at the equivalent (β-) face of the pyrroline/dihydrothiazine rings, suggesting that a common stereoisomer is favored for high-affinity interactions with L1. Furthermore, these data indicate that L1-catalyzed cephem hydrolysis can occur without either loss of the 3′ leaving group or protonation at N-5, expanding the range of mechanisms by which L1 inactivates diverse β-lactam antibiotics. These results can be exploited in developing antibiotics resistant to the action of dizinc MBLs, as well as potent MBL inhibitors, by exploring novel compounds that form long-lasting complexes.

## Results

### Steady-state kinetics

The originally identified L1 variant from *S. maltophilia* IID 1275 catalyzes the hydrolysis of a wide variety of bicyclic β-lactam antibiotics, including those from the carbapenem, penem, cephem, and penicillin classes (41, 42, 43). Like all studied MBLs, L1 is unable to hydrolyze the monocyclic monobactam aztreonam (42). We initially extended earlier kinetic studies to investigate L1-catalyzed hydrolysis of a wider range of carbapenem antibiotics as well as the unusual penam mecillinam. The results show that L1 manifests little selectivity between penems and carbapenems with different substituents at C-2, or for the presence/absence of a β-methyl group at the carbapenem C-1 position (Fig. 1), with all those substrates tested exhibiting a catalytic efficiency (*k*_cat_/*K*_M_) greater than 0.8 s^−1^/μM^−1^ (Table 1). The cephems moxalactam (a 7α-methoxy oxacephem), cefmetazole, and cefoxitin (7α-methoxy cephalosporins) all exhibit similar *K*_M_ values, indicative of their structural similarities, but moxalactam turnover is at least 15-fold slower than that of cefmetazole and cefoxitin (Table 1). Ceftazidime, an oxyimino cephalosporin with a bulky C-7 substituent, has a significantly higher *K*_M_, resulting in a *k*_cat_/*K*_M_ approximately 100-fold lower than that for the other cephalosporins (41, 42) and four-fold lower than that of moxalactam (Table 1). The *k*_cat_/*K*_M_ values for the penams, mecillinam, and penicillin G are similar, with both having relatively high catalytic efficiencies and turnover rates (Table 1).

**Table 1:** L1 steady state kinetic data

Substrate	Antibiotic class	[Enzyme] (nM)	*K*_M_ (μM)	*k*_cat_ (s^−1^)	*k*_cat_/*K*_M_ (s^−1^/μM^−1^)	Reference
Meropenem	1β-m Carbapenem	10	16.1 (1.50)	28.8 (0.68)	1.79	This study
Imipenem	Carbapenem	8	37.2 (4.80)	79.1 (3.0)	2.13	This study
Ertapenem	1β-m Carbapenem	10	13.9 (2.40)	23.8 (0.94)	1.72	(43)
Biapenem	1β-m Carbapenem	10	31.4 (1.90)	30.1 (0.50)	0.958	This study
Doripenem	1β-m Carbapenem	10	5.15 (0.86)	14.7 (0.39)	2.86	This study
Tebipenem	1β-m Carbapenem	10	18.8 (1.67)	30.7 (0.57)	1.64	This study
Panipenem	Carbapenem	10	6.16 (1.37)	5.23 (0.35)	0.849	This study
Faropenem	Penem	10	36.3 (8.80)	43.7 (2.70)	1.20	(44)
Moxalactam	Oxacephem	NR	5 (1)	0.15 (0.01)	0.030	(41)
Cefmetazole	7α-m Cephalosporin	NR	4.1 (0.2)	4.7 (0.1)	1.10	(41)
Cefoxitin	7α-m Cephalosporin	NR	3.3 (0.4)	2.2 (0.1)	0.67	(41)
Ceftazidime	Cephalosporin	500	260 (NR)	1.7 (NR)	0.007	(42)
Mecillinam	Penam	10	75.8 (12.5)	146 (8.60)	1.92	This study
Penicillin G	Penam	NR	75 (10)	410 (20)	5.5	(41)

Errors of the mean in parentheses (n = 3).

1β-m indicates presence of a 1β-methyl group. 7α-m: 7α-methoxy cephalosporin.

Abbreviations: NR, not reported.

### X-ray crystal structures of hydrolyzed antibiotics bound to L1

**Figure 2:**
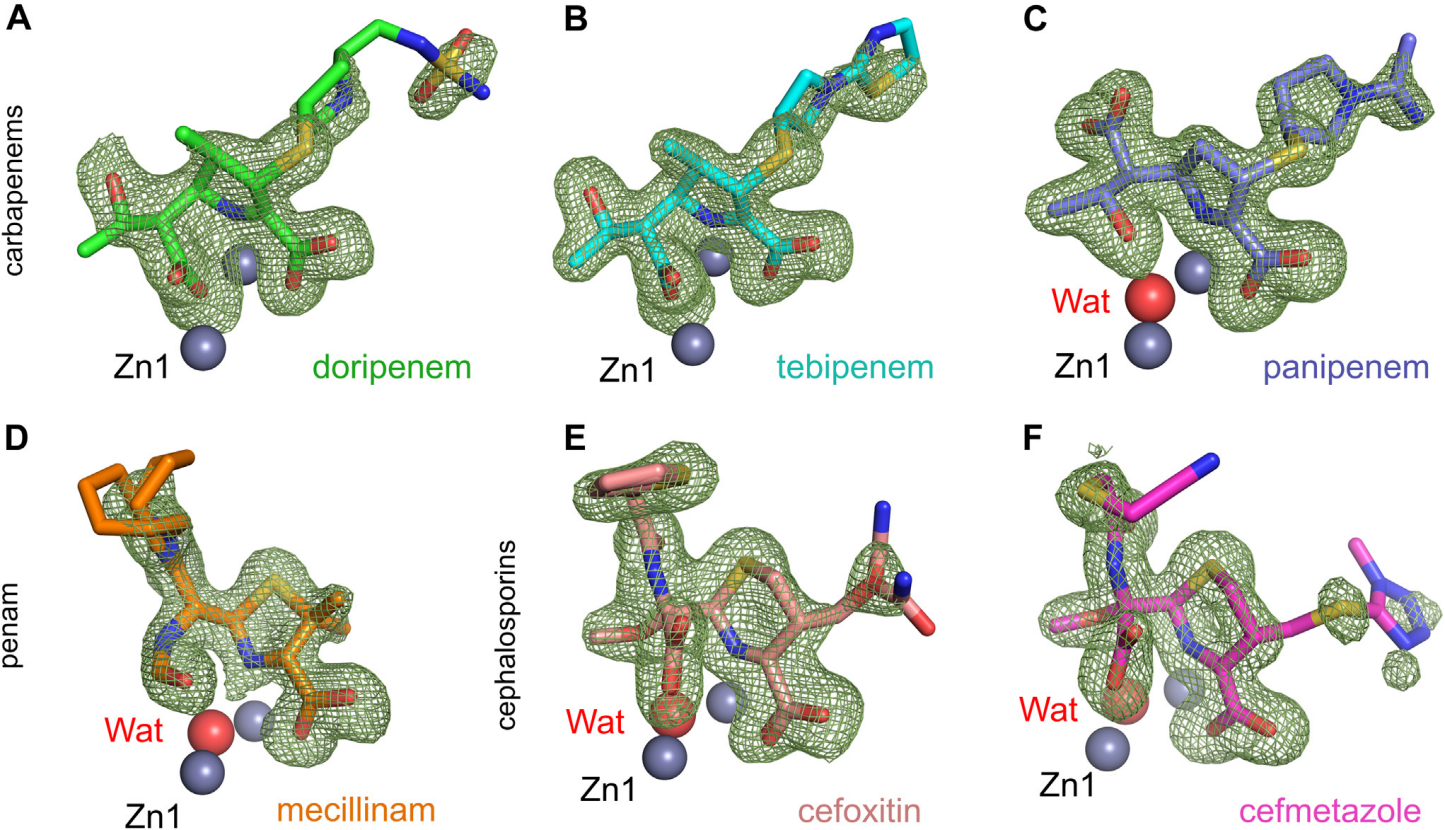
**Hydrolyzed β-lactam antibiotics bound to L1.***F*_o_-*F*_c_ electron density maps (*green mesh*) calculated from the final refined structure after removal of ligand are shown contoured at 3σ (carbapenems and penam) or 2.8σ (cephalosporins). Hydrolyzed products (see Fig. S3 for chemical structures) are modeled for (*A*) doripenem (*green*), (*B*) tebipenem (*cyan*), (*C*) panipenem (*purple*), (*D*) mecillinam (*orange*), (*E*) cefoxitin (*salmon*), (*F*) cefmetazole (*pink*). Model bias was removed through the calculation of electron density maps after five rounds of refinement (in Phenix) of the structure in the absence of a ligand.

To investigate the interactions made by β-lactam antibiotics with L1, we soaked preformed L1 crystals with solutions of all classes of β-lactams, followed by flash-cooling at various time points (30 min to 48 h) and collected X-ray diffraction data at resolutions extending to 1.43 to 1.63 Å (Table 2). Analysis of the electron density at the active sites implied the presence of complexes formed from six different hydrolyzed β-lactams from three classes (Fig. S1), all of which interact with the dizinc motif at the active site. The complexed products derived from tebipenem (after a 16 h soak), panipenem (8 h), doripenem (16 h), mecillinam (16 h), cefoxitin (23 h), and cefmetazole (22 h) could all be confidently modeled into *F*_o_-*F*_c_ electron density (Fig. 2). Experiments with other antibiotics using the same procedure either produced no electron density in the active site or density that could not be clearly interpreted as an identifiable product.

**Table 2:** Crystallographic data collection and refinement statistics for L1 crystals with β-lactam-derived products

β-lactam class	Carbapenems			Penam	Cephalosporins	
β-lactam	Doripenem	Tebipenem	Panipenem	Mecillinam	Cefoxitin	Cefmetazole
PDB accession	7ZO2	7ZO3	7ZO4	7ZO5	7ZO6	7ZO7
Data collection						
Beamline	DLS I03	DLS I03	DLS I03	DLS I24	DLS I03	DLS I03
Space group	*P*6_4_22	*P*6_4_22	*P*6_4_22	*P*6_4_22	*P*6_4_22	*P*6_4_22
Molecules/ASU	1	1	1	1	1	1
Cell dimensions						
a, *b*, *c* (Å)	105.43, 105.43, 98.18	105.27, 105.27, 98.24	105.08, 105.08, 98.42	105.61, 105.61, 98.45	105.35, 105.35, 97.89	105.54, 105.54, 98.08
α, β, γ (°)	90.0, 90.0, 120.0	90.0, 90.0, 120.0	90.0, 90.0, 120.0	90.0, 90.0, 120.0	90.0, 90.0, 120.0	90.0, 90.0, 120.0
Wavelength (Å)	0.97623	0.97623	0.97623	0.975	0.97628	0.97633
Resolution (Å)	66.86–1.49 (1.52–1.49)	66.83–1.43 (1.46–1.43)	66.82–1.43 (1.45–1.43)	49.22–1.43 (1.45–1.43)	66.74–1.61 (1.64–1.61)	91.40–1.63 (1.67–1.63)
*R*_pim_	0.015 (1.425)	0.016 (1.138)	0.014 (1.514)	0.023 (1.832)	0.016 (1.058)	0.024 (0.705)
CC 1/2	1.00 (0.563)	1.00 (0.305)	1.00 (0.364)	0.999 (0.355)	1.00 (0.566)	0.999 (0.667)
*I*/σ*I*	22.3 (0.4)	18.0 (0.2)	19.1 (0.2)	14.2 (0.5)	21.3 (0.4)	17.6 (1.6)
Completeness (%)	100.0 (100.0)	99.2 (97.5)	100.0 (98.7)	100.0 (99.8)	100.0 (99.9)	100.0 (100.0)
Redundancy	38.4 (37.4)	38.8 (39.6)	38.5 (39.1)	38.0 (36.9)	35.5 (36.1)	37.9 (38.1)
Refinement						
Resolution (Å)	66.86–1.49	66.83–1.43	66.82–1.43	46.53–1.43	45.62–1.61	66.86–1.63
No. reflections	52,777	56,733	55,502	59,974	41,940	40,673
*R*_work_/*R*_free_	17.64/20.46	17.32/19.71	16.26/20.0	15.85/17.89	16.40/18.80	15.90/17.47
#. non-H atoms						
Protein	1999	2028	2008	2035	2010	2042
Solvent	237	256	255	314	183	233
Zn ions	2	2	2	2	2	2
Ligand	28	26	24	46	58	31
B-factors						
Protein	37.8	35.2	37.5	31.9	40.9	29.7
Solvent	44.5	44.8	46.5	44.7	46.4	38.4
Zn ions	31.2	27.8	28.2	25.0	33.2	23.8
Ligand	80.6	45.9	41.9	48.4	49.7	58.0
R.m.s. deviations						
Bond lengths (Å)	0.010	0.009	0.009	0.009	0.010	0.008
Bond angles (°)	1.072	0.998	1.040	1.062	1.052	1.010
Ramachandran (%)						
Outliers	0.00	0.00	0.00	0.00	0.00	0.00
Favoured	96.20	96.59	96.21	96.59	96.97	96.21

Values in parentheses are for high-resolution shell.

The products derived from mecillinam and cefoxitin could both be modeled with two conformations, resulting from the flexibility of their C-6 or C-3 substituents (R/R_2_ side chains, Fig. 1), with refined occupancies of 0.17/0.83 and 0.29/0.71, respectively. The final refined models resulted in real space correlation coefficients (as calculated by the protein data bank [PDB] (44)) of 0.94/0.94 (mecillinam), 0.91/0.91 (cefoxitin), 0.93 (cefmetazole), 0.95 (doripenem), 0.92 (tebipenem), and 0.93 (panipenem). All of the β-lactam derived products are bound in the dizinc active site, in a manner apparently stabilized by binding in a pocket containing four hydrophobic residues (Trp39, Phe160, Ile166, and Pro227 (Fig. S2); standard MBL numbering is used throughout (19)) that are conserved amongst known L1 variants (39, 40). Below we describe the identified product complexes from each of the different classes. Fig. S3 shows detailed schematics of the binding modes, including interaction distances, of all products.

### The binding mode of carbapenem-derived products is apparently determined by the 1β-methyl group

**Figure 3:**
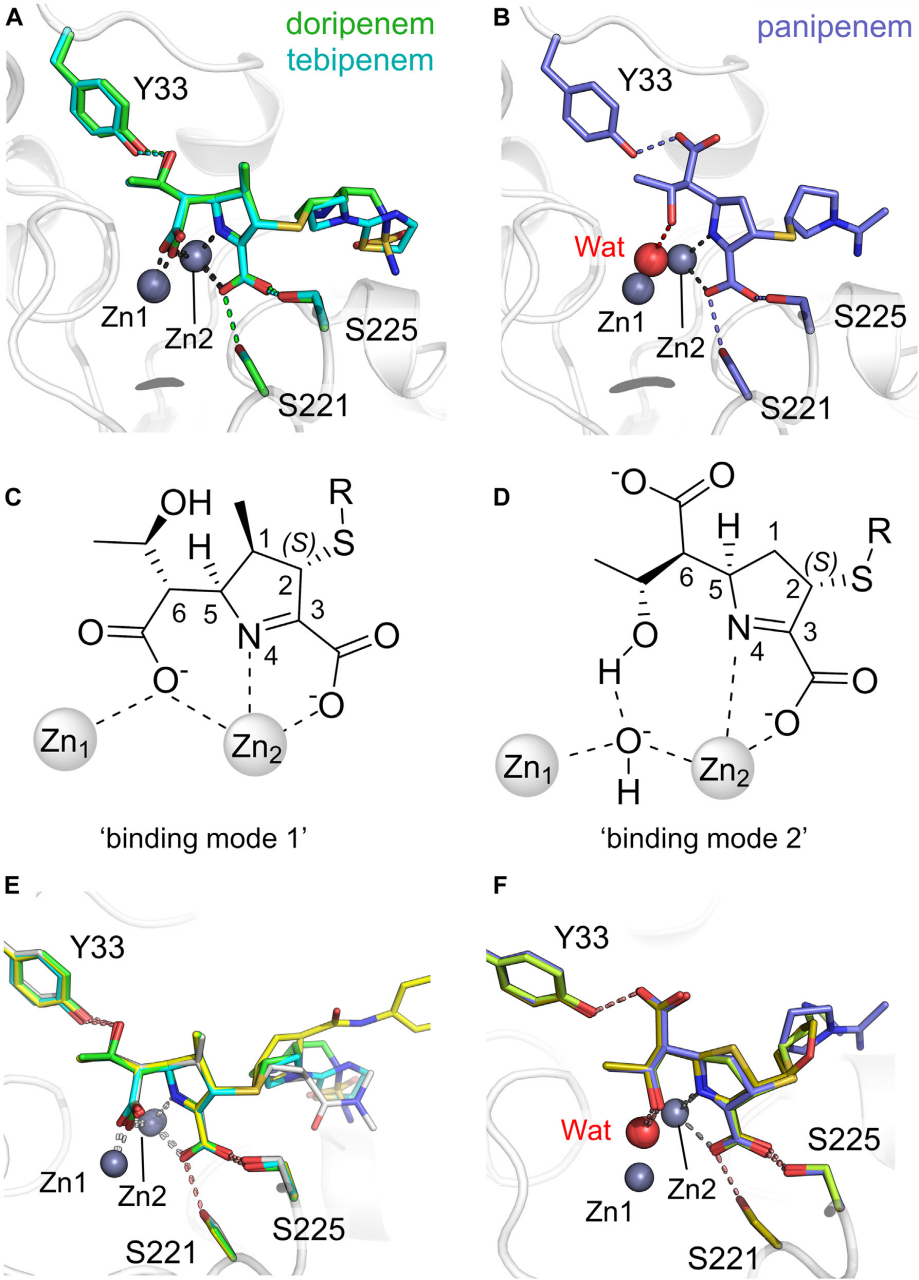
**The binding modes of carbapenem-derived products to L1.** Views from the active sites, with Zn ions shown as *gray spheres*. *A*, overlay of doripenem- (*green*) and tebipenem-derived (*cyan*) complex views. *B*, the panipenem-derived complex retains a Zn-bridging water/hydroxide (Wat, *red sphere*). *C*, schematic of “binding mode 1” in which the C-6 carboxylate of the carbapenem-derived product displaces the Zn-bridging hydroxide. *D*, “binding mode 2” in which the C-6 hydroxyethyl group interacts with the Zn-bridging hydroxide. *E*, overlays of derived products in binding mode one for tebipenem (*cyan*), doripenem (*green*), and ertapenem (PDB 7O0O (43), *yellow*) complexed to L1 and meropenem (PDB 6UAH (46), *grey*) to L1c. *F*, overlays of derived products in binding mode 2: L1:panipenem (*purple*), L1:faropenem (PDB 7A63 (47), *gold*), and L1c:imipenem (PDB 6UAF (46), *lime*).

Like most MBLs, L1 is an extremely efficient carbapenemase that hydrolyzes multiple carbapenem antibiotics containing diverse C-1 and C-2 substituents. We characterized the binding to L1 of the hydrolyzed products of tebipenem and doripenem, which contain a 1β-methyl group, and panipenem, which has a β-hydrogen atom at C-1. All three products are apparently bound (at least predominantly) as their Δ^1^-imine tautomers, with C-2 *sp*^3^ hybridized and in the (*S*)-configuration. All of the respective C-2 substituents (R_2_-groups in Figs. 1 and S1) are directed toward the solvent and do not interact with the protein main chain, consistent with similar catalytic efficiencies across the carbapenem class. The tebipenem- and doripenem-derived products bind almost identically (Fig. 3*A* and S3), with the C-6 carboxylate (formed on opening of the β-lactam ring) bridging the Zn ions, apparently replacing the catalytic water/hydroxide. This results in an increase in the Zn1 - Zn2 distance from 3.5 Å (unliganded L1, PDB 1SML (45)) to 3.8/4.1 Å for doripenem/tebipenem, respectively. The C-6 α-hydroxyethyl substituent is therefore rotated away from the Zn ions and is positioned to hydrogen bond with Tyr33. The pyrroline C-3 carboxylate interacts with Zn2 and with the side chain oxygen atoms of Ser221 and Ser225; the pyrroline N further interacts with Zn2. By contrast, while the pyrroline core of hydrolyzed panipenem makes similar interactions with Zn2 and Ser221/225, binding differs due to the rotation of the C-6 carbon that results in its hydroxyethyl group interacting with the Zn-bridging water and the C-6 carboxylate hydrogen bonding with Tyr33 (Figs. 3*B* and S3). In the complex with hydrolyzed panipenem, the Zn - Zn distance is maintained at 3.6 Å, which is very close to the value observed in uncomplexed L1 (PDB 1SML (45)).

The three carbapenem-derived structures presented here are thus representative of two binding modes which describe the interactions of hydrolyzed carbapenems with subclass B3 MBLs (Fig. 3, *C* and *D*), including with an L1 variant (from *S. maltophilia* K279a, referred to as L1c in (39), 92% sequence identity with L1) for which no kinetic data are available. In binding mode 1 (Fig. 3*E*), as observed in complexes of hydrolyzed tebipenem, doripenem, and ertapenem (PDB 7O0O (43)) with L1, and of hydrolyzed meropenem with L1c (PDB 6UAH (46)), the Zn-bridging hydroxide is displaced, resulting in an increase in the Zn - Zn distance. In binding mode 2 (Fig. 3*F*), represented by L1:panipenem (this work), L1c:imipenem (46) and L1:faropenem (a penam, PDB 7A63 (47)), the bridging hydroxide is retained and the Zn - Zn distance is as observed in the uncomplexed enzyme.

Taken together, these results suggest that the precise binding mode of (hydrolyzed) carbapenems is determined by a combination of the presence/absence of the carbapenem 1β-methyl group and the presence of Tyr33 in L1. In particular, with the 1β-hydrogen carbapenems (imipenem, panipenem) interaction of the C-6 carboxylate with Tyr33 (binding mode 2) is made possible by a rotation (about the C-5 - C-6 bond) that would otherwise be sterically hindered by a β-methyl at C-1. As a consequence, 1β-methyl-carbapenems (doripenem, tebipenem, ertapenem) adopt binding mode one in interactions with L1. The proposed role of Tyr33 is supported by comparisons with carbapenem complexes of the related subclass B3 MBL SMB-1 (48), which lacks the extended N-terminus of L1 (and thus does not contain Tyr33). In these structures the C-6 carboxylate is not constrained by Tyr33 and can rotate away from the 1β-methyl group, allowing hydrolyzed meropenem to adopt binding mode 2 with SMB-1 (Fig. S1). We have previously noted, based on quantum mechanics/molecular mechanics (QM/MM) simulations of faropenem- and ertapenem-derived products bound to L1, that it is unlikely that the two forms could interconvert when bound to the enzyme (43). Binding modes one and two could therefore represent a stable complex formed after a hydrolyzed product has left the active site and subsequently rebound to the enzyme after accumulation in solution.

### A rearranged mecillinam degradation product can bind to the L1 active site

**Figure 4:**
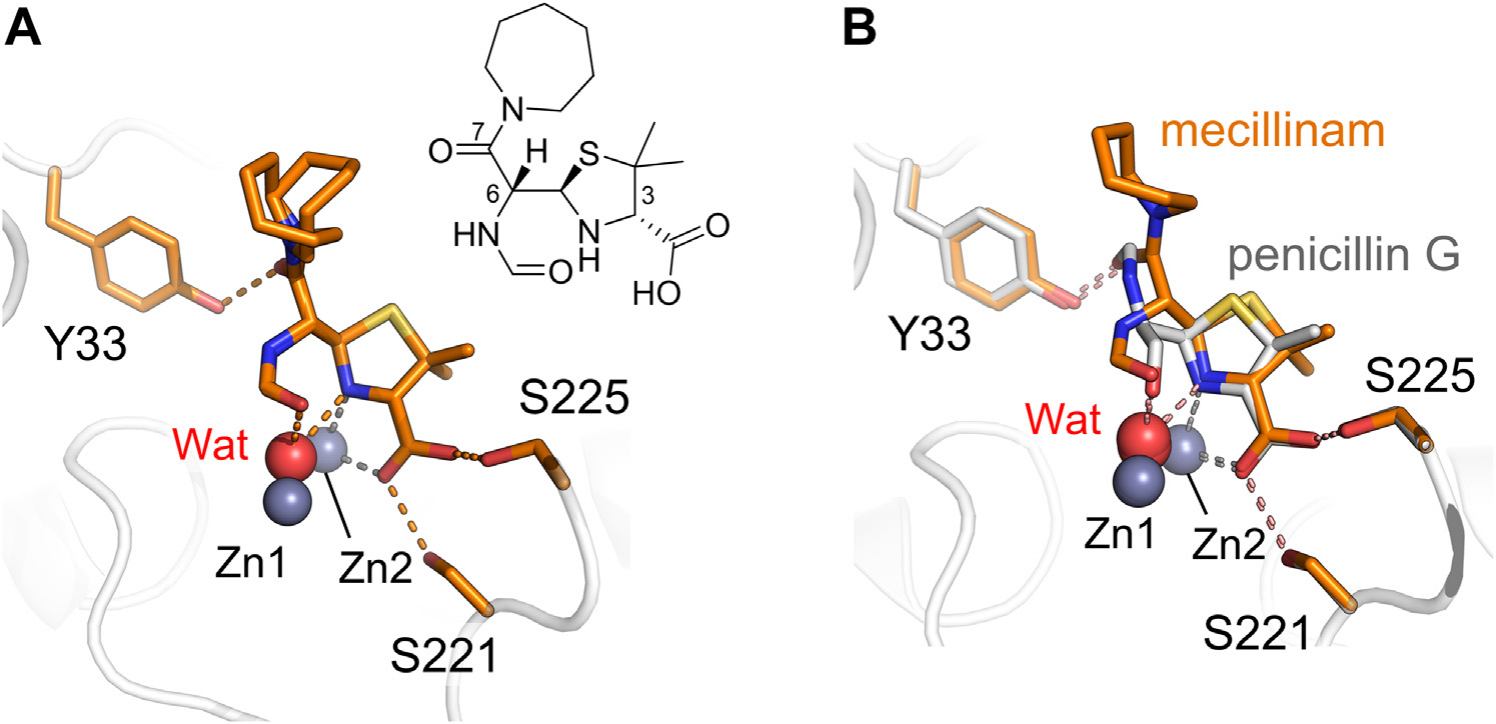
**Interactions of penam products with L1.** Views from the active sites of (*A*) the rearranged mecillinam degradation product (a penicilloic amide, *orange*) bound to L1 (*inset*, chemical structure of the modelled product); (*B*) overlay of the mecillinam degradation product in L1 with an undefined penicillin G product (hydrolysis or degradation) bound to L1c.

Mecillinam is a penam antibiotic developed in the 1970s (49). Soaking L1 crystals with mecillinam yielded electron density that clearly defined a major bound product in the L1 active site, which could not be modeled as the anticipated penicilloic acid (Fig. 1*B*). However, a penicilloic N-formyl amide, previously identified as a minor rearranged degradation product of mecillinam breakdown in solution (50), was a good fit to the electron density (Fig. 4*A*). To support our assignment of this species as the best explanation of our crystallographic data, the products of mecillinam degradation catalyzed by L1 in solution were investigated by NMR spectroscopy. A time series of one-dimensional ^1^H-NMR experiments identified the mecillinam hydrolysis product as slowly rearranging (over a time scale of hours to days) to multiple species. Prolonged (2 week) incubation followed by semi-preparative HPLC enabled the isolation of the four major observed products (Figs. S5–S8 and S13–S29). The identities of these were confirmed by one- and two-dimensional NMR experiments, enabling their assignment as the enzyme-catalyzed hydrolyzed penicilloic acid (compound **1**), its non-enzymatically formed epimer (**2**), and both epimers of the non-enzymatically formed rearranged penicilloic N-formyl amide (**3** and **4**). Of these, the penicilloic N-formyl amide (**4**) is observed bound in the crystal structure and is the only product to satisfactorily fit the experimental electron density. These data also show that the rearranged penicilloic N-fromyl amide can form from the enzyme-catalyzed penicilloic acid. The crystallographically observed product (**4**) contains a newly formed N-formyl amide at C-6 that is positioned 3 Å from the Zn-bridging water/hydroxide (see Fig. S3 for a detailed schematic of the interactions, including distances). Hydrogen bonds form between the C-7 carbonyl (of the C-6 azepane-containing side chain) and Tyr33 as well as between the thiazolidine C-3 carboxylate and Ser221/Ser225. It would seem likely that the complex with **4** is formed after initial L1-catalyzed hydrolysis of mecillinam followed by rearrangement in solution and subsequent rebinding.

A penicillin G-derived product has previously been observed by crystallography at the active site of the L1 variant, L1c (PDB 6U0Z (46)). Although labeled and discussed as a penicilloic acid, in this structure many atoms, including the entirety of the C-6 side chain as well as an oxygen atom from the expected C-6 carboxylate, could not be modeled due to the absence of electron density. However, the binding mode for this product to L1c is similar to that for the mecillinam-derived penicilloic N-formyl amide observed here for L1 (Fig. 4*B*). Thus, although no known penicillin G breakdown products seem to fit the electron density in L1c, it is possible that the observed product in PDB 6U0Z is a mixture of different species (resulting in poorly defined electron density) or a yet-to-be-identified penicillin-derived rearrangement product, rather than a penicilloic acid. We note that rearranged penam degradation products have not previously been observed crystallographically complexed with B1 MBLs, with the expected penicilloic acid (**1**) being well-defined as a complex with NDM-1 (51, 52).

### An intact 3′ leaving group in cephem-derived complexes with L1

**Figure 5:**
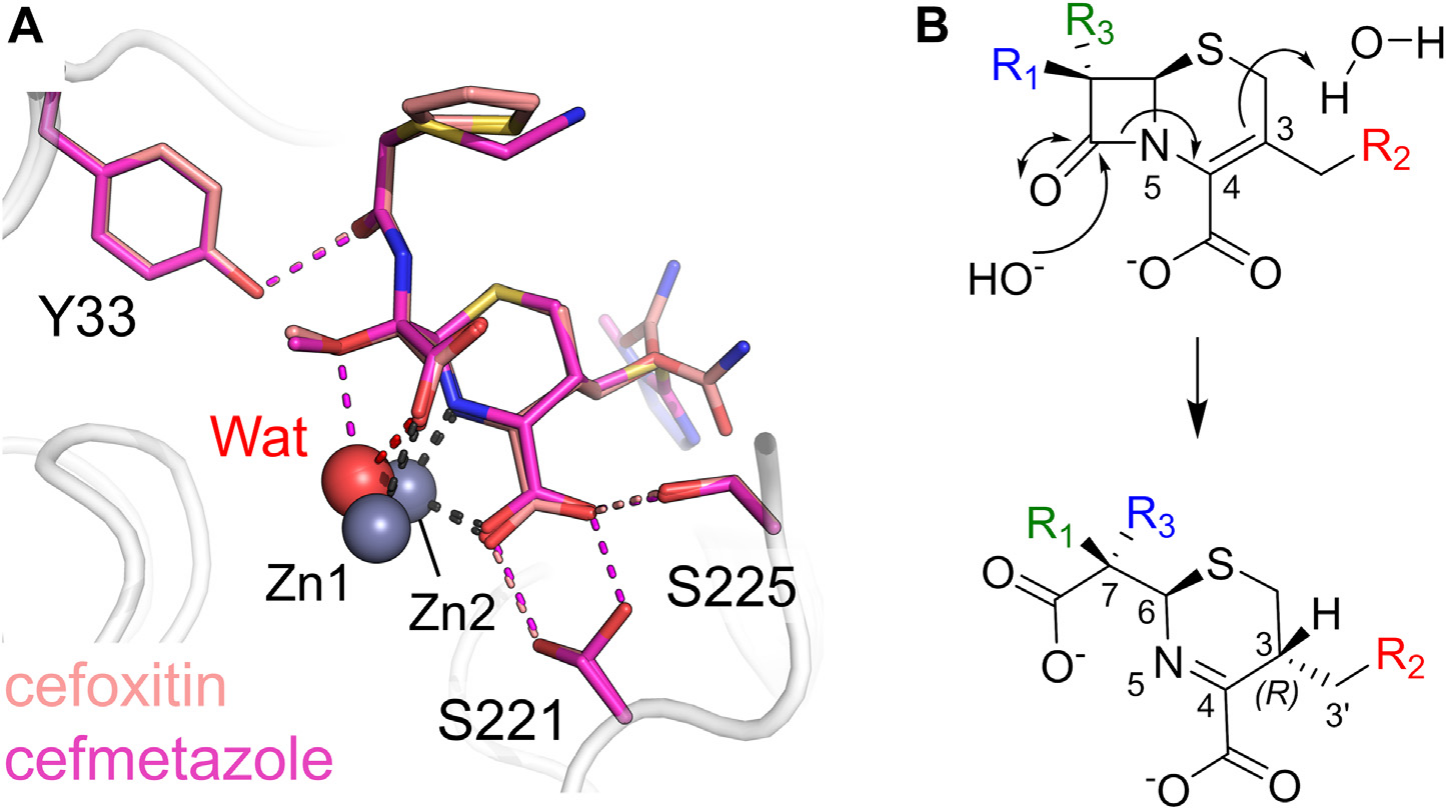
**Cephem-derived products bound to L1.***A*, views from the active sites of cefoxitin- (*salmon*) and cefmetazole-derived (*pink*) products in L1. *B*, simplified possible pathway to the formation of the crystallographically observed products involving protonation of C-3 (resulting in 3R stereochemistry) from the same face of the dihydrothiazine ring as in carbapenems.

Both cefoxitin and cefmetazole contain a 7α-methoxy substituent (R_3_ group, green in Fig. S1). Their hydrolyzed products bind to L1 in an almost identical fashion, with the Zn-bridging water present and interacting with the C-7 methoxy (R_3_) and carboxylate groups (Fig. 5*A*). This binding mode causes a slight decrease in the distance between Asp120 and the Zn-bridging water from 2.8 Å (PDB 1SML (45)) to 2.6 Å. There is also a small increase in the Zn - Zn distance, to between 3.6 and 3.7 Å from the 3.5 Å observed in the uncomplexed enzyme (PDB 1SML (45)), consistent with EXAFS data obtained with the cephem nitrocefin (53) and with the structure of L1 complexed with hydrolyzed moxalactam (PDB 2AIO (23)). The C-7 carboxylate (formed on hydrolysis of the β-lactam amide) interacts with Zn2 and the bridging hydroxide/water, while the R_3_ methoxy group also interacts with the bridging hydroxide/water. In addition, the C-4 carboxylate and dihydrothiazine N both co-ordinate Zn2 (see Fig. S3 for distances). Consistent with our observations for the mecillinam complex (above), the derived products both hydrogen bond with Ser221 and Ser225 *via* their C-3 carboxylate groups and with Tyr33 *via* the carbonyl of their C-7 (R_1_) substituents.

**Figure 6:**
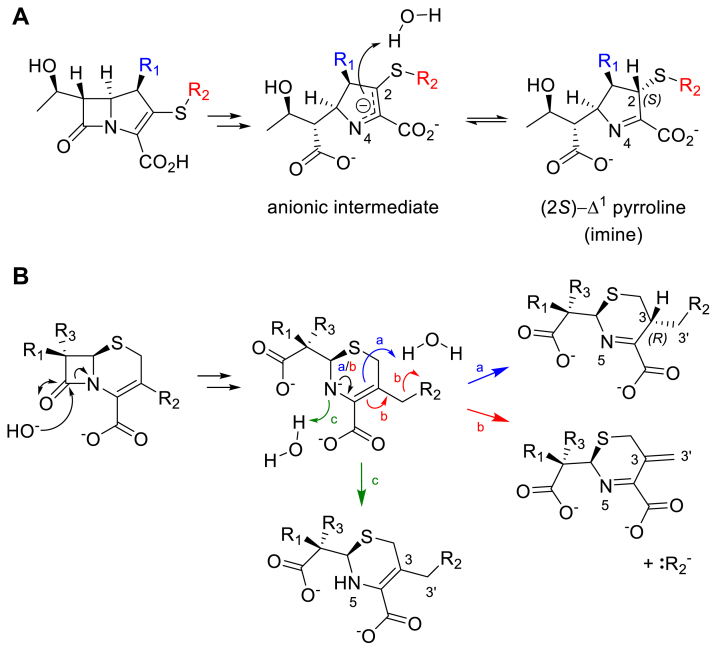
**Potential pathways for stereoselective protonation of carbapenems and cephems.***A*, possible pathway for (2*S*)-Δ^1^-imine formation in L1, *via* protonation of C-2 of an anionic intermediate by a non-activated water molecule. Note, the (2*R*)-Δ^1^-imine and Δ^2^-enamine tautomers (see Fig. 1*C*) are not observed crystallographically. *B*, cephem hydrolysis results in the formation of a common anionic intermediate (with negatively charged N-5) that reacts to give three possible products. *Blue (a)*, tautomerization of the dihydrothiazine ring (*black arrow*) and diastereoselective protonation (3*R*) of C-3 by a water molecule. *Red (b)*, dihydrothiazine tautomerization (*black arrow*) and loss of the C-3′ substituent (R_2_), resulting in an exocyclic methylene group. *Green (c)*, N-5 protonation without tautomerization.

It is generally thought that cephems with an appropriate R_2_-leaving group will lose this on hydrolysis by β-lactamases from both the SBL and the MBL classes, with subsequent formation of a C-3 = C-3′ exomethylene group (see Fig. 1*C*). However, in our studies, both cephem-derived hydrolysis products were modeled with the C-3′ leaving group intact as evidenced by the presence of electron density for the exocyclic sulfur (cefmetazole) or oxygen (cefoxitin) atoms of the C-3 (R_2_) substituents positioned outside the plane of the cephem dihydrothiazine ring. Further, electron density next to C-3, that would indicate the presence of a C-3 = C-3′ exomethylene group, is absent. Indeed, C-3 is clearly *sp*^3^ hybridized and protonated at the equivalent face of the dihydrothiazine ring as is C-2 in the pyrroline ring of carbapenem-derived Δ^1^-imine products (note that the (3*R*) cephem and (2*S*) carbapenem configurations are stereochemically equivalent). The tetrahedral nature of C-3 then dictates that the β-lactam-derived nitrogen (N-5) is present as the imine, rather than the enamine, tautomer. This suggests a mechanism of hydrolysis involving C-3 protonation by a non-activated water molecule in the active site on the β-face of the dihydrothiazine ring (as drawn in Fig. 5*B*), which is similar to the protonation of C-2 on the equivalent face of the pyrroline ring during carbapenem hydrolysis (Fig. 6*A*).

## Discussion

Our extensive crystallographic data, combined with previous studies, enable new insight into the chemistry and binding modes of hydrolyzed β-lactam antibiotics from all classes in the active site of L1. In particular, all of the observed complexes are stabilized by common interactions with a hydrophobic binding pocket and three conserved hydrogen-bonding residues (Tyr33, Ser221 and Ser225). Moreover, by expanding our knowledge of L1:β-lactam interactions, our data reveal the extent to which these are replicated in the binding of inhibitors of different classes and go some way to rationalizing the differential activities of these toward L1.

Although many MBL inhibitors (*e.g.*, boronates, thiols) displace the Zn-bridging hydroxide (21, 54, 55), there are similarities between their interactions and those of carbapenems in both of the binding modes we observe (Fig. S9). Boronates bind to NDM-1 similar to the products of 1β-methyl-carbapenems to L1 (binding mode 1), including interactions with Zn2 and displacement of the Zn-bridging hydroxide by the boronate oxygen, together with a concomitant increase in the Zn – Zn distance. The reason why boronates (*e.g.*, taniborbactam (54), QPX7728/xeruborbactam (56)) do not efficiently inhibit L1 and its variants is currently unknown, although it has been suggested that this could be due to the lack of an L3 loop in L1 (a region that in subclass B1 MBLs interacts with substrates and inhibitors of multiple classes), or of an active site residue (equivalent to Lys224 in NDM-1), able to interact with the inhibitor carboxylate (56). However, interactions involving the boronate (and substrate) carboxylates are not conserved across complexes with different B1 enzymes and in L1 could be provided by Ser221, while hydrophobic interactions with L1 (equivalent to those involving the L3 loop) could be provided by Trp39, Phe160, Ile166, and Pro227. The basis for the poor boronate potency against L1 could be that, at least in some cases, an atypical 5-coordinate, geometry is preferred at Zn2 for bicyclic boronate binding to subclass B1 enzymes, like NDM-1 (54, 57), which may be less stable in the L1 active site, in which Zn2 coordination is structurally distinct (45). In most of the L1 complexes described to date, Zn2 adopts either an octahedral (as in the antibiotic-derived complex structures presented here) or regular trigonal bipyramidal (as in complexes with indole carboxylate (InC) inhibitors (58)) geometry. Although an unusual, irregular 5-coordinate geometry was observed on bisthiazolidine binding to L1 (55), this involved loss of Zn2 coordination by Asp120, something that may be considered energetically unfavorable.

Binding mode 2 in the carbapenem-derived complexes resembles the binding of InCs with L1 (58), phosphonomethyl pyridine carboxylates (PMPCs) with the subclass B1 IMP-1 (59), and a cyclobutanone to subclass B1 SPM-1 (60) (Fig. S9*B*). All of these binding modes feature coordination of the MBL Zn2 metal ion by a carboxylate, while the Zn-bridging hydroxide interacts with either the indole N of InCs, a phosphonate oxygen of PMPCs, or a hydroxyl group of cyclobutanones. The InCs are extremely potent inhibitors of L1 (as judged by *in vitro* activities), interacting with Ser221/Ser225 in the active site without bidentate coordination of Zn2 (instead maintaining the trigonal bipyramidal geometry), and further clash, rather than make productive interactions, with L1 Tyr33 (58). Binding mode 2 in L1 is therefore most closely represented by PMPC binding to IMP-1, with the pyrroline/pyridine N and carboxylate groups forming a bidentate interaction with Zn2 (59).

The route to crystallographically observed MBL–β-lactam complexes is still incompletely understood, largely because we (and others) cannot rule out that these may result from rebinding events of hydrolyzed β-lactams, rather than representing states along the hydrolysis pathway. This is probably the case for the rearranged mecillinam degradation product, as we show that following initial hydrolysis its formation is likely non-enzymatic and possibly results from an intermolecular reaction of an intermediate with free hexamethyleneimine (or azepane, which is formed subsequent to mecillinam hydrolysis ((50) and Fig. S5)).

The possibility of complex formation through rebinding is also important when considering the crystallographically observed hydrolyzed carbapenem complexes, all of which are in the (*S*)-Δ^1^-imine tautomer. It has been suggested that diastereoselective protonation at C-2 of an anionic intermediate can occur on the enzyme to form the (*S*)-Δ^1^-imine in a mechanism for carbapenem hydrolysis by NDM-1 (22) (Fig. 6*A*). Studies on the reactions of both MBLs and SBLs with the carbapenem biapenem also reveal that the (*S*)-imine is the major product formed (31). However, evidence has been presented, including with L1, that the nascent Δ^2^-enamine can rapidly tautomerize (either on enzyme or in solution) to the (*R*)-Δ^1^-imine, which then converts slowly to the more stable (*S*)-Δ^1^-imine over longer time scales (30). In our work we observed no crystallographic evidence for the (*R*)-Δ^1^-imine, and note that its formation at the L1 active site is likely to be sterically unfavorable.

The (3*R*) cephalosporin-derived products, protonated at the β-face of their dihydrothiazine ring, are stereochemically equivalent to the (2*S*) carbapenem-derived products, highlighting that configurations of antibiotic-derived products protonated on the opposite (α-) face are less preferred and less stable in the MBL active site. Further, our previous QM/MM simulations indicate that the Δ^2^-enamine product of ertapenem was less stable at the active site than the (*S*)-Δ^1^-imine (43). Although our data therefore support the accumulation of the carbapenem-derived (*S*)-Δ^1^-imine on the active site, as previously suggested (22), two possible explanations for this exclusive observation present themselves: either the Δ^2^-enamine product rapidly leaves the L1 active site, tautomerizes in solution to the (thermodynamically favored) (*S*)-Δ^1^-imine and then rebinds or the (*S*)-Δ^1^-imine slowly accumulates in the active site, leaving more slowly than other tautomeric forms/stereoisomers (whose initial production may be preferred kinetically) due to the increased stability of the L1 complex. Recent QM/MM studies (61) on the L1 reaction pathway suggest that C-2 protonation of an imipenem-derived anionic intermediate was blocked at the active site by Pro227 on the L10 loop (Fig. S2). However, the identification of candidate proton donors requires high-resolution views of intact β-lactam (Michaelis-Menten) or intermediate complexes present earlier in the hydrolytic reaction to be definitive. Furthermore, the flexibility of the L10 loop, that was not modeled in QM/MM simulations, may allow access of water molecules to this position to facilitate protonation at the β-face of carbapenem and cephem hydrolysis products (Fig. 6).

Cephem hydrolysis (Fig. 6*B*) proceeds *via* a common anionic intermediate (in which N-5 is negatively charged) that is stabilized by interaction with Zn(II) (62, 63, 64, 65). In two possible pathways of cephem product formation, the double bond in the dihydrothiazine ring migrates and the β-lactam-derived N is not protonated (red and blue pathways in Fig. 6*B*). The resultant products differ in that the C-3′ group is either eliminated (5, 29) or is retained with C-3 protonated and in the *sp*^3^ hybridization state (as observed crystallographically here and by NMR (66)). A third possible pathway (green in Fig. 6*B*) involves protonation of the amide N of the anionic intermediate without tautomerization of the cephem dihydrothiazine (5), as suggested for substrates without a 3′ leaving group, such as the chromogenic reporter substrate nitrocefin (5, 67). Crystallographic evidence for this third pathway is limited; the C-3 atom is trigonal in a crystal structure of the acylenzyme complex of cefalexin (a cephalosporin that lacks a 3′ leaving group) with a mutant SBL (containing a noncanonical amino acid, PDB 4ZJ3 (68)); and in two structures of class D (OXA) SBLs complexed with hydrolyzed ceftazidime (PDB 6Q5F (69) and PDB 4X55 (70)), most crystal structures of SBL acylenzyme complexes lack the C-3′ group. The product of this third pathway has not been observed in NMR studies of cephalosporin reaction products formed on hydrolysis by MBLs (29, 66, 71) This suggests that particularly for cephalosporins with a ‘good’ C-3′ leaving group, β-lactamase-catalyzed cephem hydrolysis N protonation is less favored than the migration of dihydrothiazine double bond and elimination of the 3′ substituent (27), although this is likely dependent on both the enzyme and the properties of the C-3 substituent (R_2_ group) (5, 27).

The crystallographically observed cephem species described here are clearly stable for many hours bound to L1, indicating it is unlikely they readily degrade to the C-3′ eliminated product, at least in crystals. Instead, these seem to be the endpoint of an alternative pathway for MBL-catalyzed cephem hydrolysis that involves protonation at C-3, at the β-face of the dihydrothiazine ring (as drawn in Figs. 5*B* and 6), rather than at N-5. In L1, candidate proton donors to C-3 could be water molecules situated close to Ser225 in the unliganded structure (PDB 1SML (45)) or that interact with the intact C-3 substituent (Fig. S10). In the subclass B1 enzyme NDM-1, water molecules coordinated by the L10 loop (PDB 3SPU (72)) appear well positioned to perform this role, with previous molecular dynamics simulations and QM/MM simulations confirming their presence in this position to potentially facilitate C-2 protonation at the equivalent (β-) face of the pyrroline ring of bound carbapenems (22, 61). Our crystallographic observations with cephems and carbapenems, together with previous findings, indicate that a common, diastereoselective mechanism for protonation at C-3/C-2 of chemically diverse cephalosporins and carbapenems may exist for MBLs from different classes.

A similar cefuroxime-derived product (from a co-crystal structure, PDB 5O2E (45, 52)), with C-3 in a tetrahedral *sp*^3^ configuration and therefore likely protonated, has also been observed bound to NDM-1 (Fig. S11). It was suggested that this was an anionic intermediate (*i.e.*, with C-3 negatively charged) formed prior to the loss of the C-3′ leaving group (71). However, this might be expected to be highly unstable (especially over the course of an extended co-crystallization experiment) and solution NMR studies imply rapid loss of the C-3′ leaving group on the reaction of cefuroxime with NDM-1 (71). A species, derived from a novel cephalosporin, that features a tautomerized dihydrothiazine ring (containing a N-5=C-4 double bond) and intact C-3′ group has also been observed as a potentially inhibitory complex formed on hydrolysis by the subclass B1 MBL IMP-1 (66).

Oxacephem (moxalactam) hydrolysis by L1, which is 100-fold less efficient than that of the 7α-methoxy cephalosporins cefoxitin and cefmetazole, likely differs as a crystal structure shows loss of the R_2_ group at C-3 after a 90 min soak (23), reflecting the final product from the reaction shown in Figure 1*C*. In contrast to what we describe here, there is no evidence of *sp*^3^ hybridization at C-3 during moxalactam hydrolysis, suggesting that the pathway in Figure 5*B* for cephalosporins may not be preferred for oxacephems. However, interactions made by moxalactam- and cefoxitin-derived products are almost identical (Fig. S12), regardless of the presence of the C-3′ group.

## Conclusion

Our crystallographic results reveal that L1 manifests preferential binding of species formed after diastereoselective protonation, at C-2/C-3, on the β-faces of the pyrroline and dihydrothiazine rings of carbapenems and cephalosporins, respectively (Fig. 6). Our observation of such cephem-derived products, *sp*^3^ hybridized at C-3, that retain their C-3′ leaving groups, implies that for L1-catalyzed hydrolysis of at least some cephems, C-3 protonation of an anionic intermediate, *via* a non-activated water molecule in the active site, is preferred to N protonation by a Zn-bound water. It is then possible that a similar pathway may operate in carbapenem hydrolysis by L1 and other MBLs such as NDM-1. How the initial intact β-lactam binds (*i.e.*, formation of the nascent non-covalent active site complex) is yet to be established, with the crystal structures herein likely snapshots of hydrolysis endpoints. However, our results indicate that multiple β-lactam-derived hydrolysis and/or degradation products can bind to the L1 active site and importantly establish the most stable complexes and species. This information can be exploited to design compounds able to form long-lived species at the active site of dizinc MBLs such as L1, able to function either as small molecule MBL inhibitors or as novel antimicrobials resistant to MBL-catalyzed degradation. Such entities will be particularly important in counteracting AMR mediated by subclass B3 MBLs, such as L1, that currently evade the activity of the most potent, and closest to the clinic, MBL inhibitors.

## Experimental procedures

### Crystallization and structure determination

Recombinant L1 from *S. maltophilia* strain IID 1275 (41, 73) was expressed and purified as previously described (55). L1 was crystallized at 4 °C: 2 μl protein (23 mg/ml in 10 mM Tris pH7, 100 mM NaCl, 5 mM ZnSO4, 1 mM β-mercaptoethanol) was mixed with 2 μl crystallization reagent [0.1 M HEPES pH 7.75, 2% PEG400, 1.9 M NH_4_(SO_4_)_2_] in ChrysChem24 sitting drop plates (Hampton Research). Crystals grew within 3 weeks and were soaked by placing them into cryo-solution (crystallization reagent plus 25% glycerol) supplemented with antibiotics from time points ranging from 30 min to 48 h before flash-cooling in liquid nitrogen. Data were collected and structures determined from crystals soaked in 18 mM tebipenem for 16 h, 12.5 mM doripenem for 16 h, 18 mM panipenem for 8 h, 10 mM mecillinam for 16 h, 15 mM cefoxitin for 23 h, and 20 mM cefmetazole for 22 h. Diffraction data were collected at Diamond Light Source on beamlines I24 (mecillinam) and I03 (all other soaks). Data were processed using the xia2 pipeline at Diamond Light Source (74) and integrated in XDS (75) (mecillinam) or Dials (76) (all others) and scaled in XSCALE (75) (mecillinam), Dials (cefmetzole, cefoxitin, panipenem, tebipenem) or in Aimless in the CCP4 suite (77) (doripenem).

Phases were solved using molecular replacement in Phaser (78), with PDB 7O0O (43), with the ligand removed, as the starting structure. Ligand geometries were calculated in eLBOW in Phenix (79) and modelled into *F*_o_-*F*_c_ density. Structures were completed with iterative rounds of refinement in Phenix and Coot (80) and validated by Molprobity (81) and Phenix. Figures were created in PyMol (http://www.pymol.org/, 82).

### Steady state kinetics

Enzyme assays were performed at 25 °C in 10 mM HEPES pH 7.5, 150 mM NaCl, 0.02 mM ZnSO4, and 50 μg/ml bovine serum albumin in Greiner half area 96-well plates, and a Tecan Infinite 200 pro microplate reader. Steady state kinetic parameters were calculated by measuring initial rates of β-lactam hydrolysis. Kinetic parameters were calculated and analyzed using the Michaelis-Menten curve in GraphPad Prism 6 (GraphPad Software, La Jolla, CA, USA; www.graphpad.com). The following wavelengths and extinction coefficients were used: tebipenem (Δε_297_ = −9550 M^−1^ cm^−1^ (83)); panipenem (Δε_297_ = −7400 M^−1^ cm^−1^ (84)); doripenem (Δε_296_ = −7540 M^−1^ cm^−1^ (85)); mecillinam (Δε_240_ = −1100 M^−1^ cm^−1^ (86)); imipenem (Δε_300_ = −9000 M^−1^ cm^−1^ (87)); meropenem (Δε_293_ = −7600 M^−1^ cm^−1^ (87)); biapenem (Δε_293_ = −8630 M^−1^ cm^−1^ (87)).

### Turnover assays of mecillinam using NMR spectroscopy

Purified L1 was buffer exchanged into phosphate buffer (50 mM phosphate buffer, pH 7.4) using microspin columns (BioRad) and concentrated using Amicon Ultra Centrifugal Filters (0.5 ml, MWCO: 10 kDa). Experiments were performed in 5 mm regular NMR tubes at 298 K. Mecillinam (100 mM stock in H_2_O or D_2_O, final concentration: one or 4 mM) was dissolved in 25 mM Tris-d_11_ in D_2_O, pD eight, or in 25 mM Tris-d_11_ in H_2_O, pH 7.5 (final sample volume was 450 μl and contained 10% (v/v) D_2_O). Hydrolysis of mecillinam was achieved after the addition of L1 (30 μM stock, final concentration: five or 50 nM). NMR spectra were recorded immediately after the addition of the β-lactamase.

NMR spectra were obtained using a Bruker AVIII HD 600 equipped with a BB-F/H Prodigy N_2_ CryoProbe, a Bruker AVIII 700 MHz NMR spectrometer (both Chemistry Research Laboratory, Oxford) or a Bruker AVIII 750 MHz NMR spectrometer (Department of Biochemistry, Oxford) equipped with an inverse 5 mm TCI ^1^H cryoprobe. Carr-Purcell-Meiboom-Gill (CPMG) NMR spectra were recorded by applying the PROJECT-CPMG sequence (88). The experimental parameters were as follows: total echo time, 40 ms; relaxation delay, 2 s. Water suppression was achieved by pre-saturation. ^1^H NMR spectra were processed with 3 Hz Lorentzian line broadening using MestReNova 14.1 (MestReLabs, Spain; www.mestrelab.com) and TopSpin 3.6.1 (Bruker, Germany; www.bruker.com).

### Isolation of compounds by HPLC and NMR analysis

Mecillinam (7.5 mg, 5 mM in 25 mM Tris-d_11_ in D_2_O, pD 8.0) and L1 (20 μl of 30 μM in 50 mM phosphate buffer, pH 7.4) were incubated for 2 weeks at room temperature. NMR spectra were recorded to measure the extent of the reaction. The products were isolated using a Shimadzu HPLC system, equipped with a SunFire semiprep column (C_18_, 5 μm, 150 mm length, 10 mm diameter). Elution employed a gradient of acetonitrile in water (with water and acetonitrile supplemented with formic acid, 0.1% v/v): 0 to 12 min (0.5 %), 12 to 47.5 min (0.5–60%), 47.5 to 50 min (60%), 50 to 52 min (60–98%), 52 to 55 min (98%), 55 to 57 min (98–0.5%), and 57 to 60 min (0.5%). The isolated compounds were lyophilized to remove the solvent and dissolved in D_2_O for NMR analysis.

## Data availability

For all crystal structures presented herein, coordinates and structure factors have been deposited to the Worldwide PDB under accession codes 7ZO2 (doripenem soak), 7ZO3 (tebipenem soak), 7ZO4 (panipenem soak), 7ZO5 (mecillinam soak), 7ZO6 (cefoxitin soak) and 7ZO7 (cefmetazole soak).

## Supporting information

This article contains supporting information (23, 45, 48, 50, 52, 54, 58, 59, 60, 71).

## Conflict of interest

The authors declare that they have no conflicts of interest with the contents of this article.
