# Supplementary material for: Interactions of hydrolyzed β-lactams with the L1 metallo-β-lactamase: Crystallography supports stereoselective binding of cephem/carbapenem products
Source: J Biol Chem. 2023 Mar 15;299(5):104606. doi: 10.1016/j.jbc.2023.104606 (PMC10148155; doi:10.1016/j.jbc.2023.104606)
Supplement: Supplemental Figures S1–S29 [file mmc1.pdf]

Supporting Information for

Interactions of hydrolyzed β-lactams with the L1 metallo-β-lactamase: Crystallography supports stereoselective binding of cephem/carbapenem products

Philip Hinchliffe, Karina Calvopiña, Patrick Rabe, Maria F. Mojica, Christopher J. Schofield, Gary I. Dmitrienko, Robert A. Bonomo, Alejandro J. Vila, James Spencer

This file contains Figures S1 – S12, NMR spectra (Figures S13 – S29), and Supporting References.

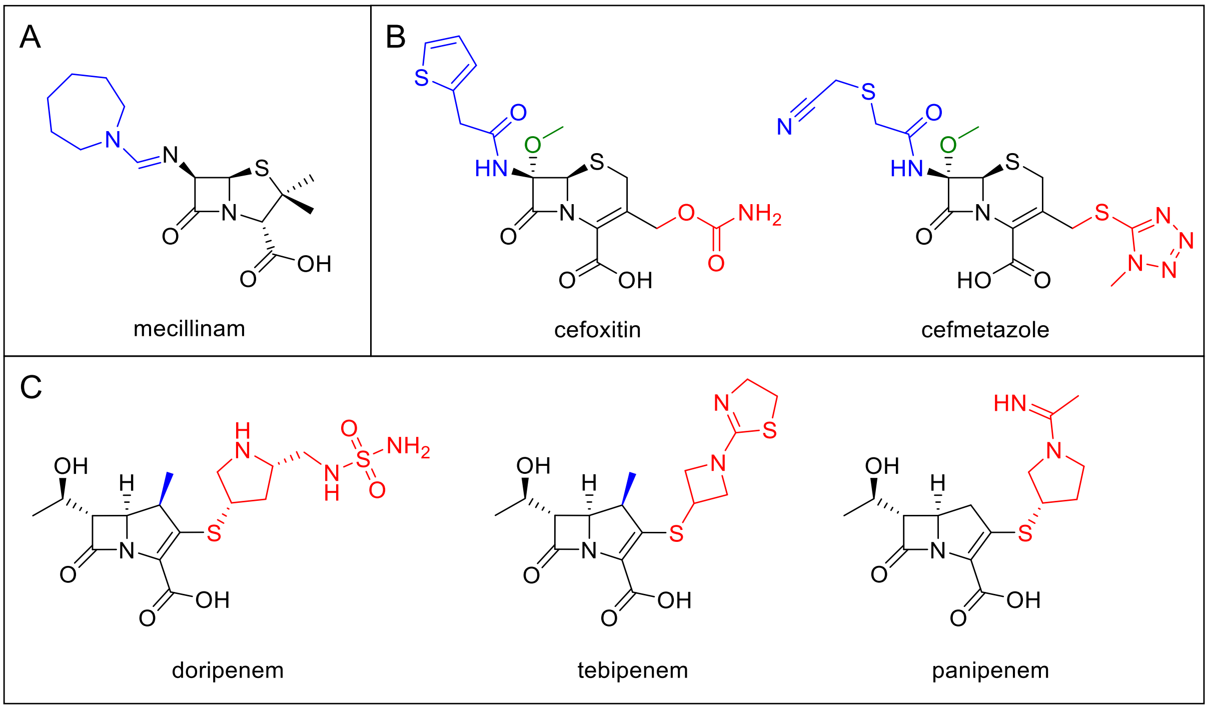

Figure S1. Bicyclic β-lactams used in the crystallographic studies. Variable R-groups are coloured as in **Figure 1A** of the main text. **(A)** Mecillinam, a penam/penicillin. **(B)** The cephems cefoxitin and cefmetazole, both of which contain a methoxy at position C-7 and so are sometimes referred to as 7α-methoxy cephalosporins. **(C)** The carbapenems doripenem and tebipenem contain a 1β-methyl group (blue), while panipenem has a 1β-hydrogen.

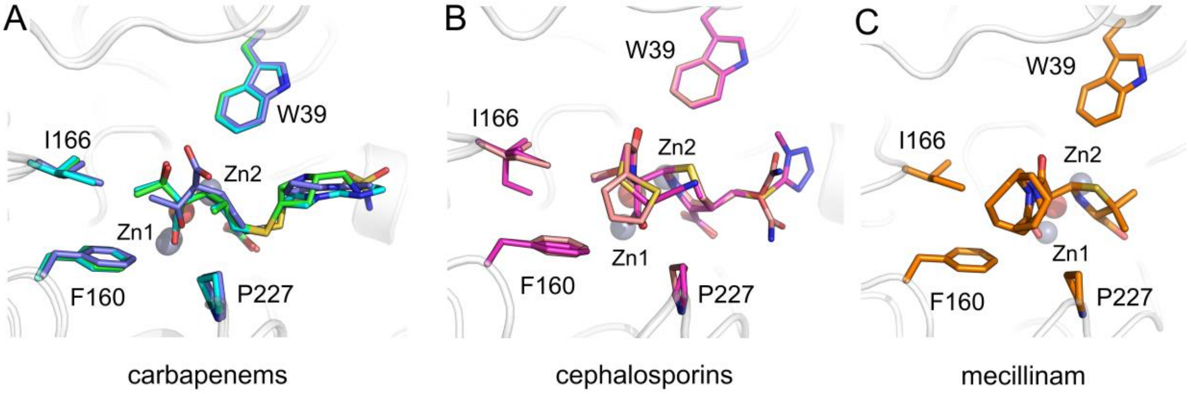

Figure S2. A common hydrophobic binding pocket. In the L1 complexes the hydrolysed antibiotics all interact with a hydrophobic pocket at L1 active site formed by the side chains of Trp39, Phe160, Ile166 and Pro227. **(A)** Overlays of the carbapenem-derived product complexes from tebipenem (cyan), panipenem (purple) and doripenem (green). **(B)** Overlays of cephalosporin-derived product complexes from cefoxitin (salmon) and cefmetazole (pink). **(C)** A rearranged mecillinam-derived degradation product complex (orange).

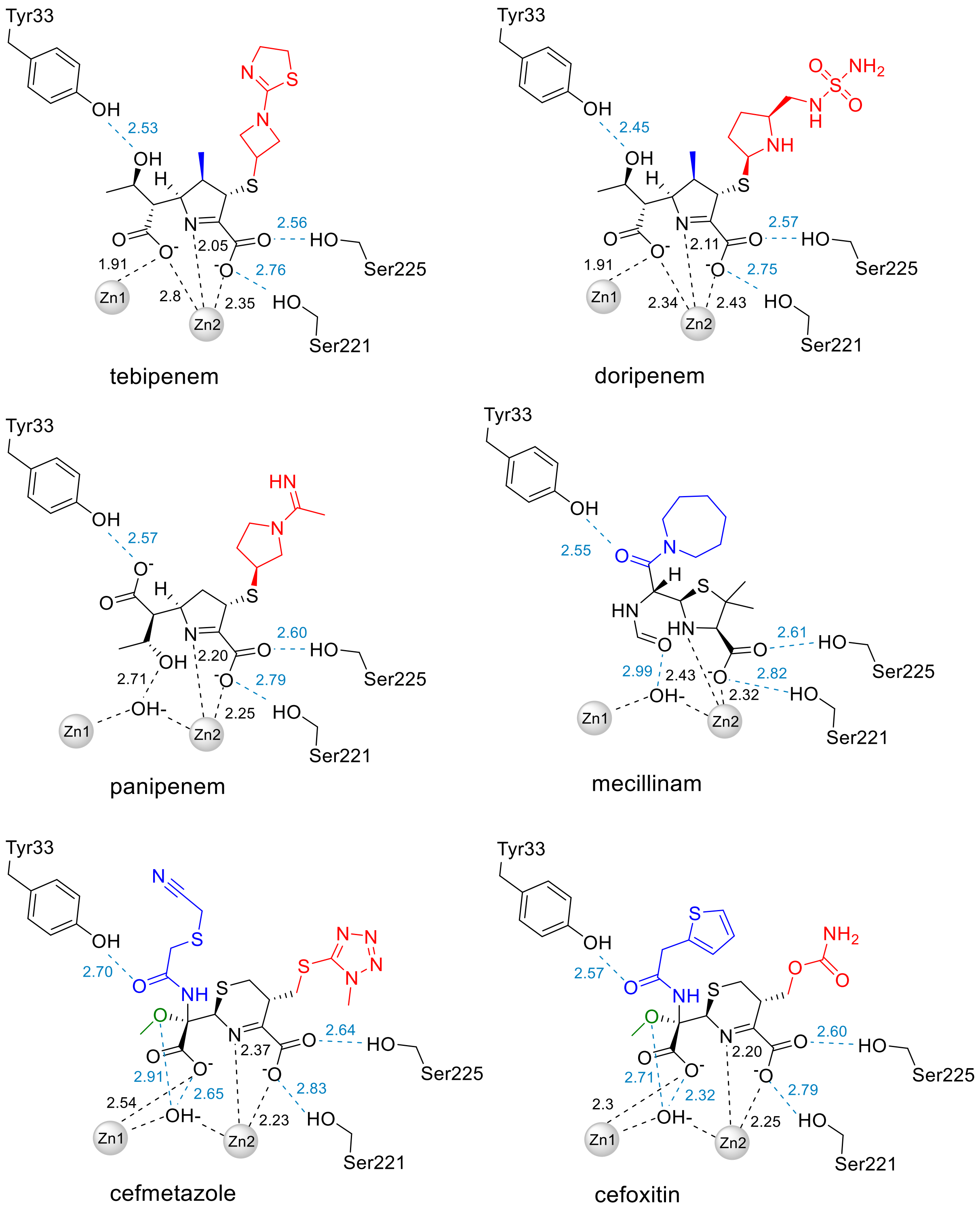

Figure S3. Schematics of the interactions of the hydrolysed antibiotics with L1. Distances (in Å) are labelled. Hydrogen bonds are shown in cyan and Zn-coordinating interactions in black. R-groups are coloured as in main text **Figure 1**. PDB codes for the L1 complexes are as follows: tebipenem-derived product, PDB 7ZO3; doripenem-derived product, PDB 7ZO2; panipenem-derived product, PDB 7ZO4; mecillam degradation product PDB 7ZO5; cefmetazole-derived product, PDB 7ZO7; cefoxitin-derived product, PDB 7ZO6.

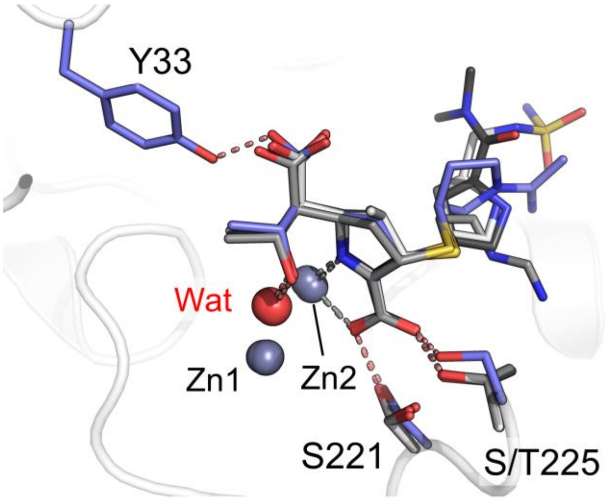

Figure S4. Binding mode 2 in the B3 MBLs SMB-1 and L1. An overlay of carbapenem-derived products bound (in binding mode 2) with the subclass B3 SMB-1 (1) (doripenem, imipenem and meropenem, shades of gray; PDB IDs 5B15, 5B1U and 5AXO, respectively) and L1 (panipenem, purple). Note, the absence of the extended N-terminus in SMB-1, and therefore of Tyr33, results in repositioning of the C-6 carboxylate in SMB-1 crystal structures and formation of binding mode 2 in carbapenems that contain a 1β-methyl, that is otherwise sterically hindered in L1.

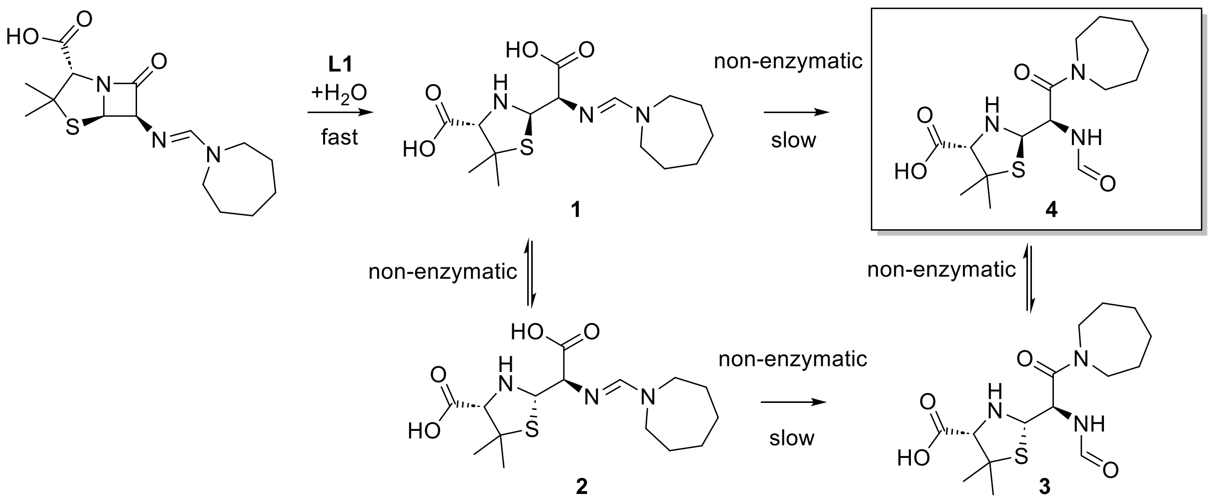

Figure S5. Hydrolysis and degradation of mecillinam. Structures of products **1** to **4** were assigned by NMR (**Figure S6**); compound **4** was also observed with L1 by crystallography (**Figure 2** and **Figure 4**, see main text). *Note*: The mechanism for the conversion from **1** to **4** and **2** to **3** has not been studied in detail but may involve reaction of the 5-*exo* trig β-lactam hydrolysis derived carboxylate with the amidine to give a lactone, followed by fragmentation to release azepane which subsequently reacts with the carbonyl of the intermediate lactone to give **3** and **4**.

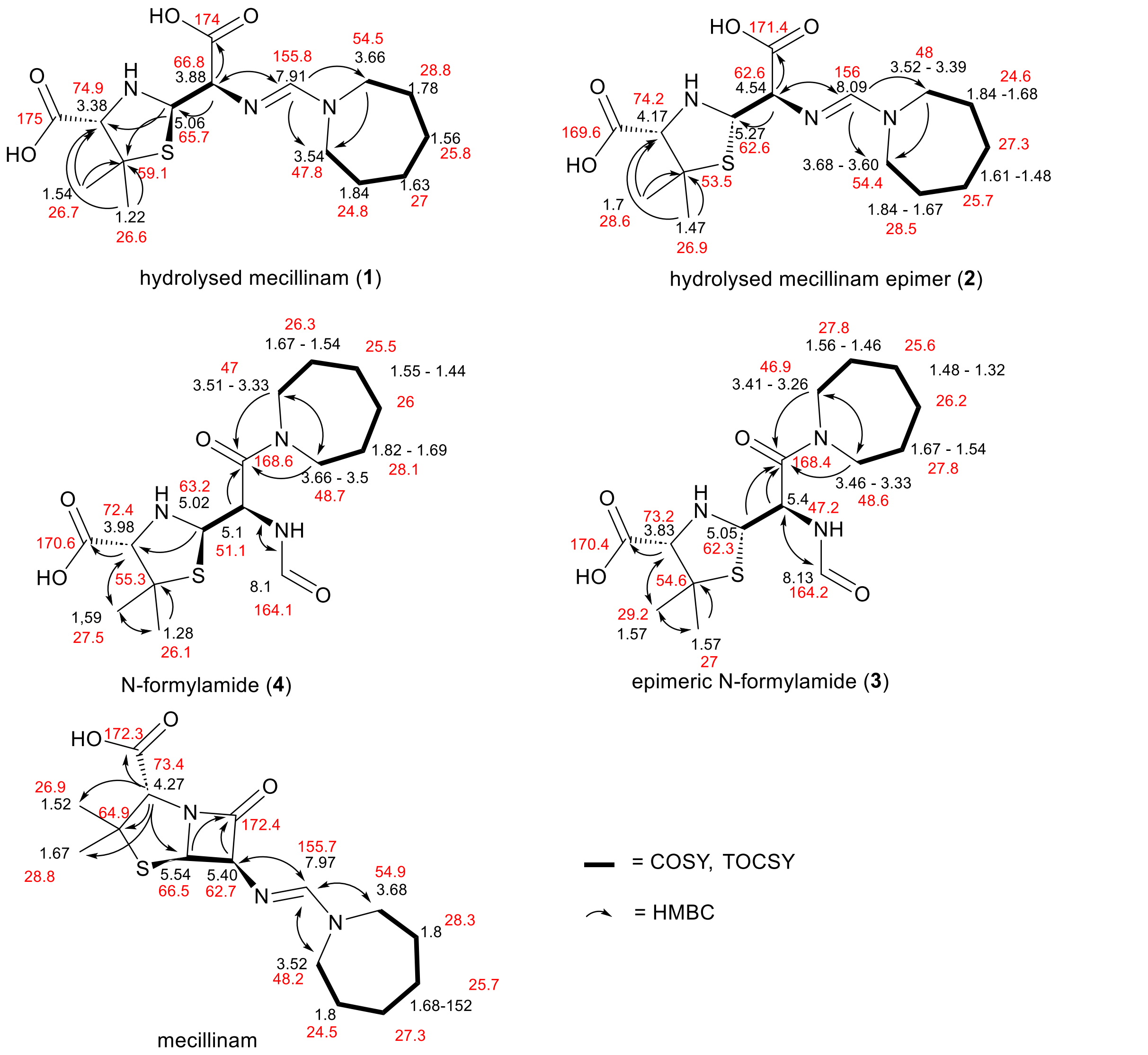

Figure S6. Assigned chemical shifts of mecillinam. The two epimers of hydrolysed mecillinam (**1** and **2**) and the two epimers of the rearranged N-formyl amide (**3** and **4**). ^{1}H chemical shifts are shown in black, ^{13}C chemicals shifts are inferred from HSQC and HMBC crosspeaks and are shown in red. COSY and TOCSY correlations are in bold, and HMBC correlations indicated with arrows. Note: The assignment of stereochemistry of **1** – **4** should be regarded as provisional, though **1** was formed as the initially found hydrolysed product.

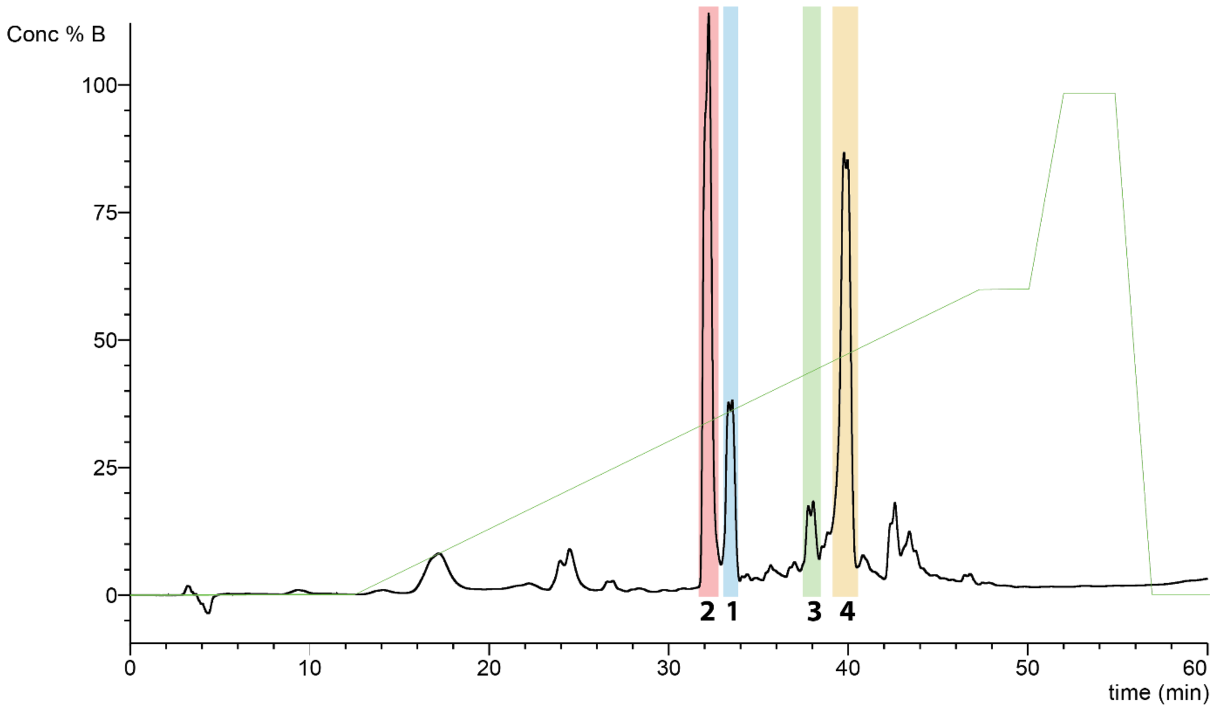

Figure S7. HPLC chromatogram of a crude extract of 5 mM mecillinam hydrolysed using L1 in 25 mM Tris-d11 in D2O, pD 8.0) after 2 weeks incubation. Isolated fractions are color-coded and numbered according to **Figure S5** with the HPLC elution gradient is in green. *Note*: Other compounds were formed during hydrolysis.

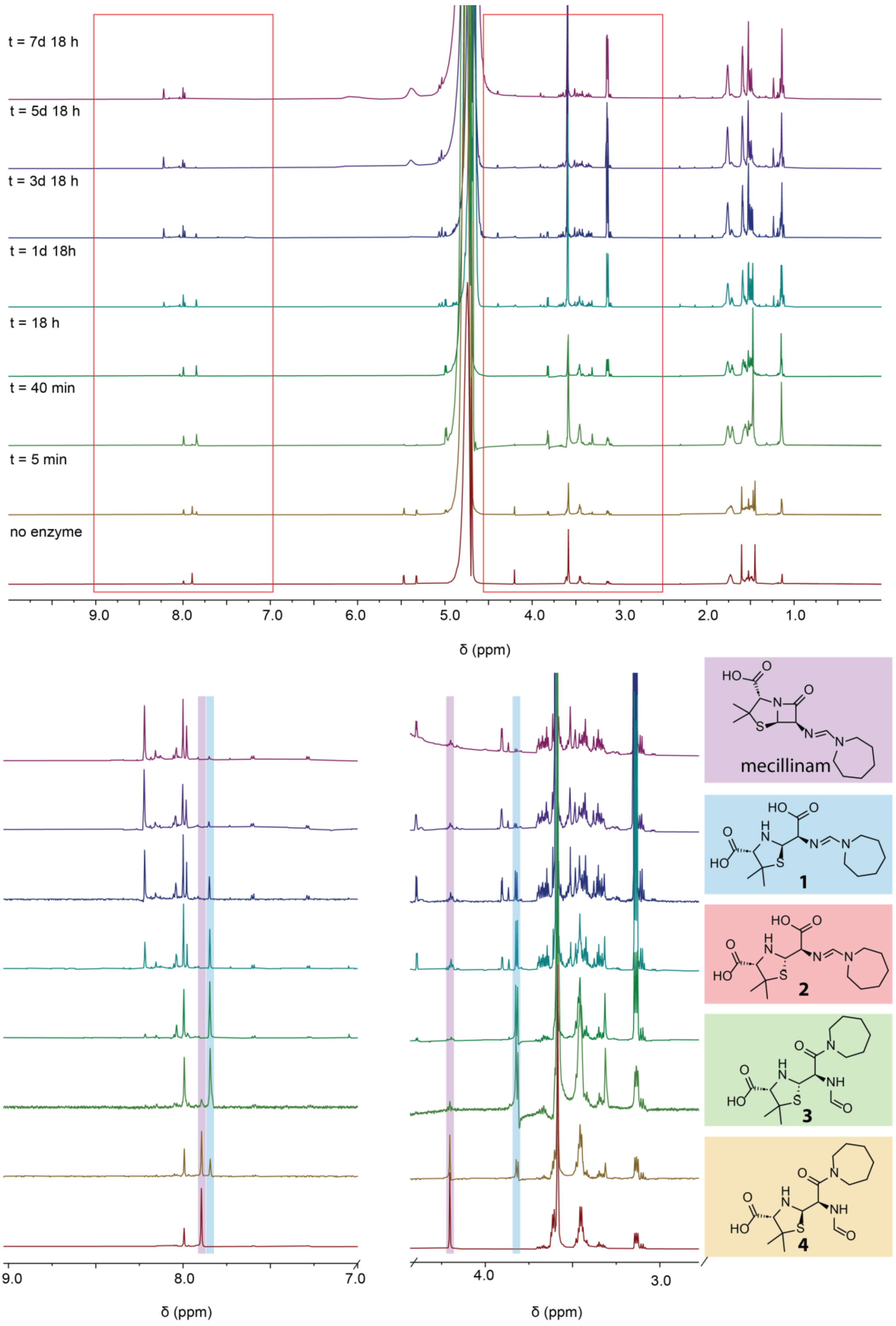

Figure S8. ^{1}H NMR time-course of the hydrolysis and degradation of mecillinam. Chracteristic peaks are highlighted in red for mecillinam and blue for the hydrolysed mecillinam, with multiple degradation/hydrolysis products forming over time.

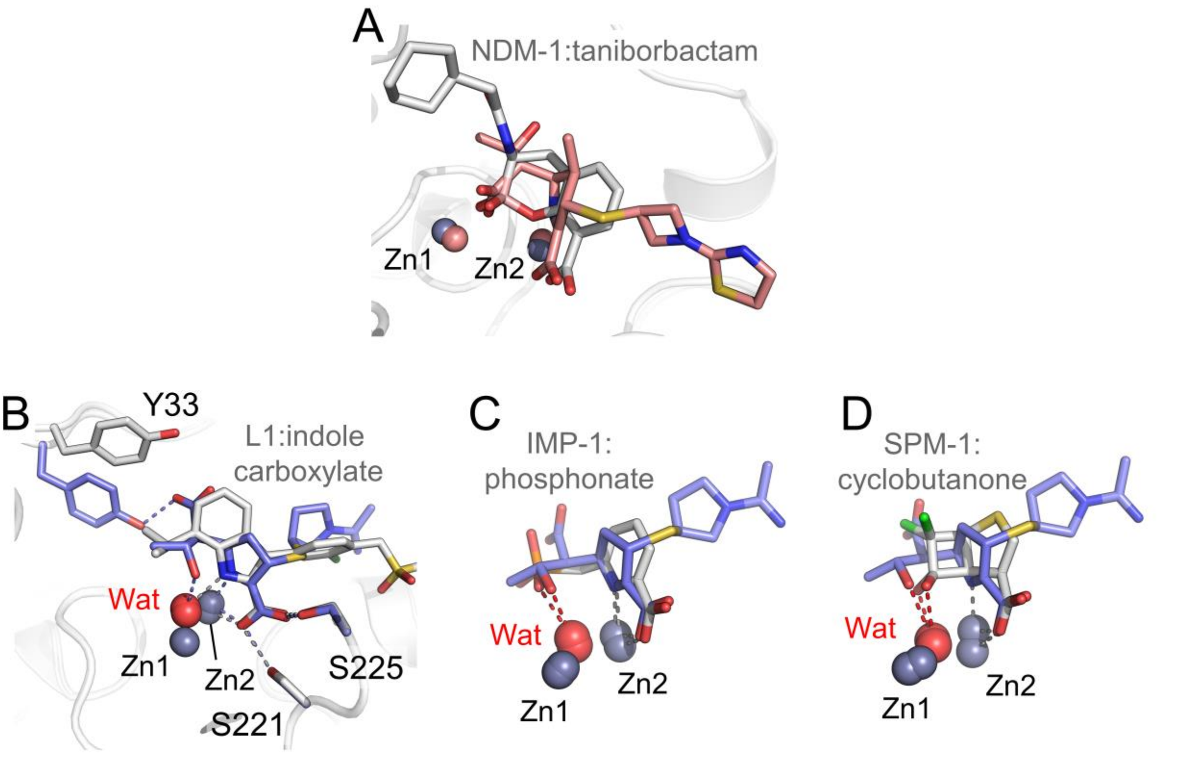

Figure S9. Comparisons of inhibitor and carbapenem-derived interactions with L1. Overlays of **(A)** taniborbactam bound to the subclass B1 MBL NDM-1 (PDB 6RMF (3), grey) and hydrolysed tebipenem bound to L1; **(B)** an indole carboxylate inhibitor (PDB 7AFZ (4), grey) and hydrolysed panipenem bound to L1. **(C)** IMP-1 with a phosphonomethyl pyridine carboxylate inhibitor (PDB 5HH4 (5), grey) and panipenem bound to L1. **(D)** A cyclobutanone inhibitor bound to SPM-1 (PDB 5NDB (6), grey) and hydrolysed panipenem bound to L1.

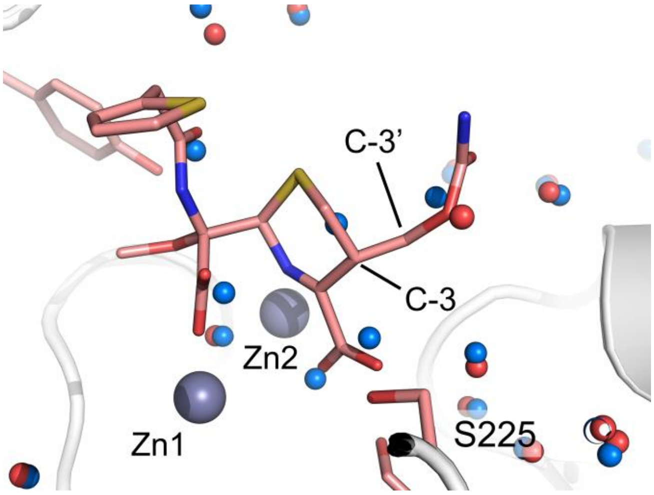

Figure S10. Water positions in unliganded and hydrolysed cefoxitin crystal structures. Views from the active sites of unliganded L1 (PDB 1SML (7)) overlaid with the crystal structure of L1 with hydrolysed cefoxitin (pink, PDB 7ZO6). Water molecules are shown as blue or red spheres for unliganded L1 or L1:cefoxitin, respectively.

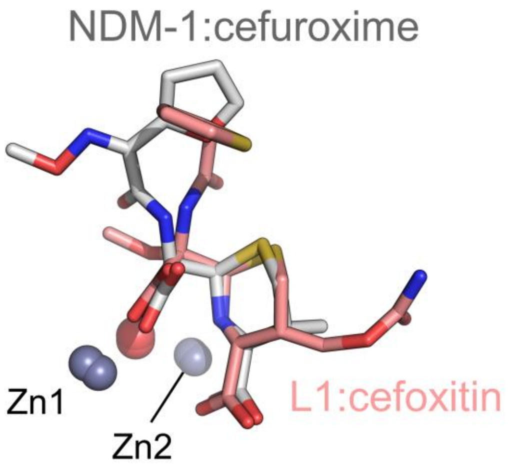

Figure S11. Comparison of the binding of cephem-derived products to L1 with NDM-1. Overlay of the active sites of L1 and NDM-1 with complexed products of cefoxitin (pink, PDB 7ZO6) and cefuroxime (grey, PDB 5O2E (8,9)), respectively.

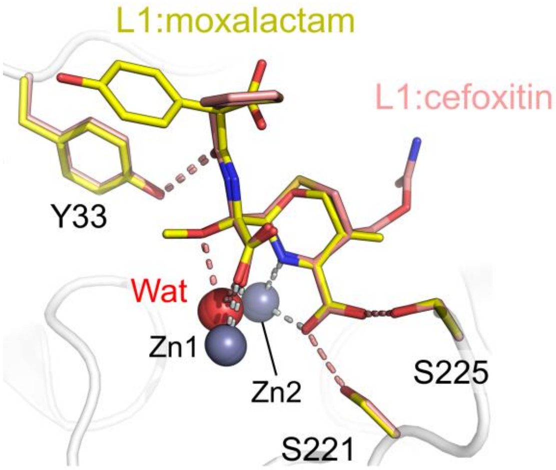

Figure S12. Hydrolysed cephems from different classes bind similarly. Overlay of L1 with hydrolysed moxalactam (PDB 2AIO (10), yellow) and cefoxitin (salmon). Both products make almost identical interactions with the protein, the zinc ions (Zn1 and Zn2, grey spheres) and the Zn-bridging hydroxide (Wat, red sphere).

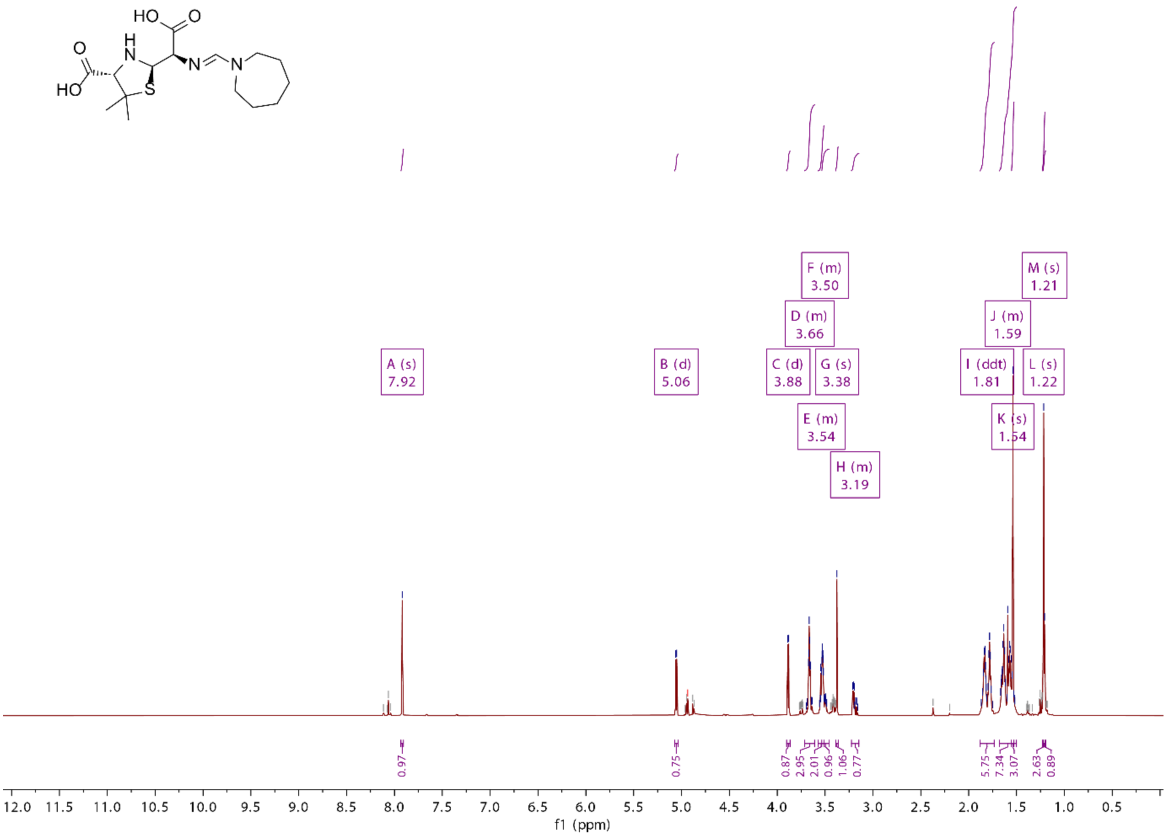

Figure S13. ^{1}H NMR spectrum (700 MHz, 25 mM Tris-d_{11} in D_2O) of hydrolysed mecillinam (**1**).

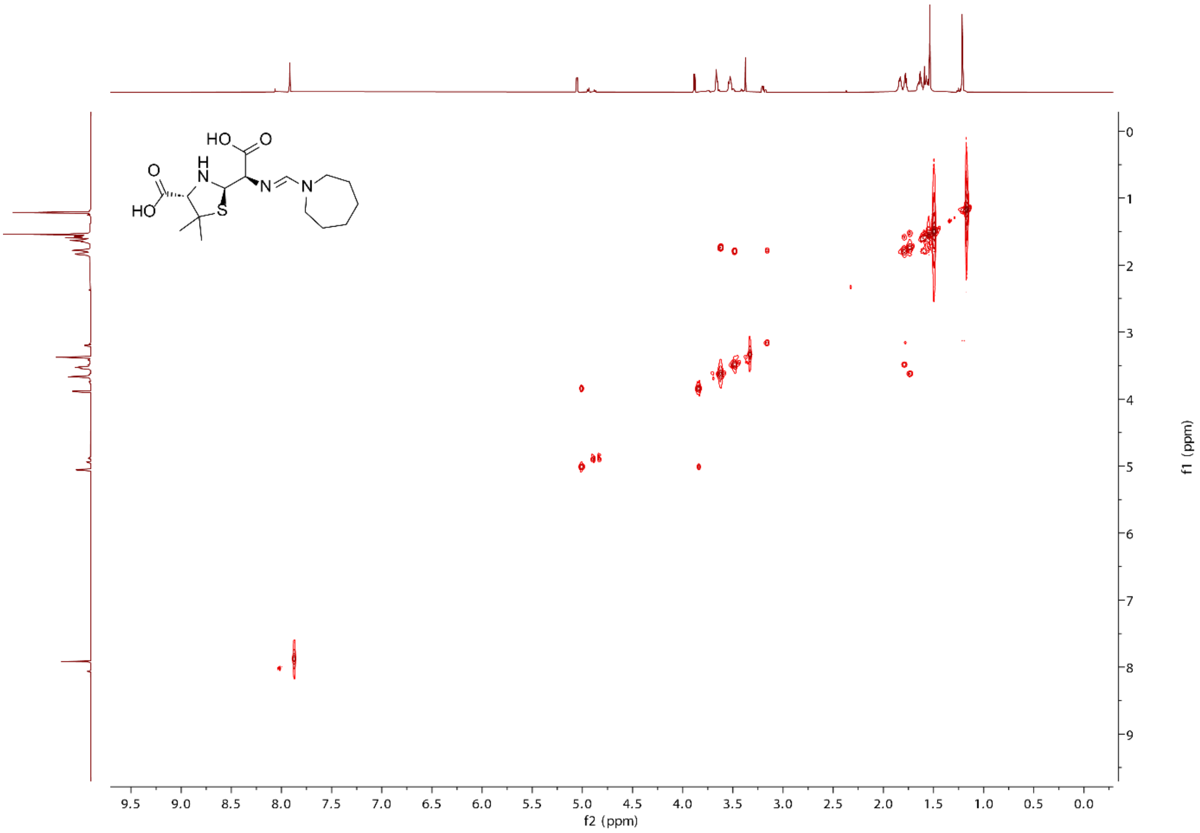

Figure S14. ^{1}H,^{1}H COSY NMR spectrum (700 MHz, 25 mM Tris-d_{11} in D_2O) of hydrolysed mecillinam (**1**). Note, the 1H projections (top and left) in the spectrum are the same as that shown in Figure S13 and are included here for easier visualisation.

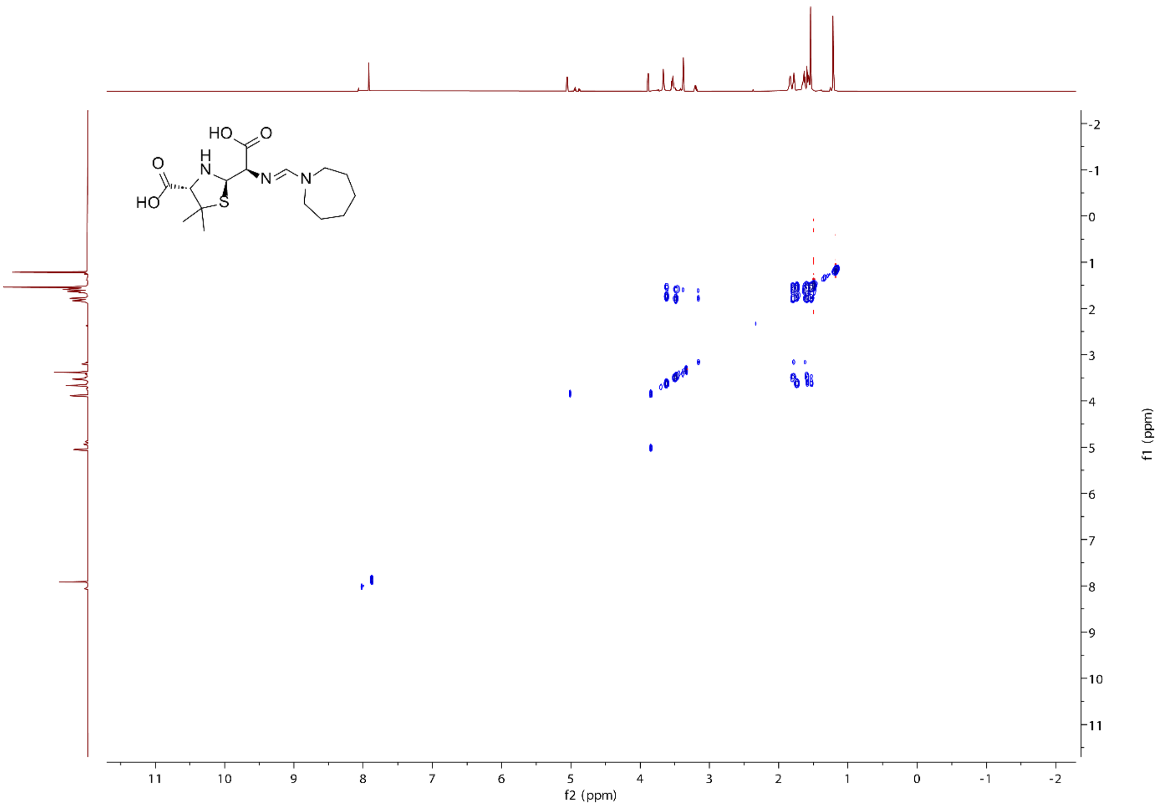

Figure S15. $^{1}H,^{1}H$ TOCSY NMR spectrum (700 MHz, 25 mM Tris-d_{11} in D_2O) of hydrolysed mecillinam (**1**). Note, the 1H projections (top and left) in the spectrum are the same as that shown in Figure S13 and are included here for easier visualisation.

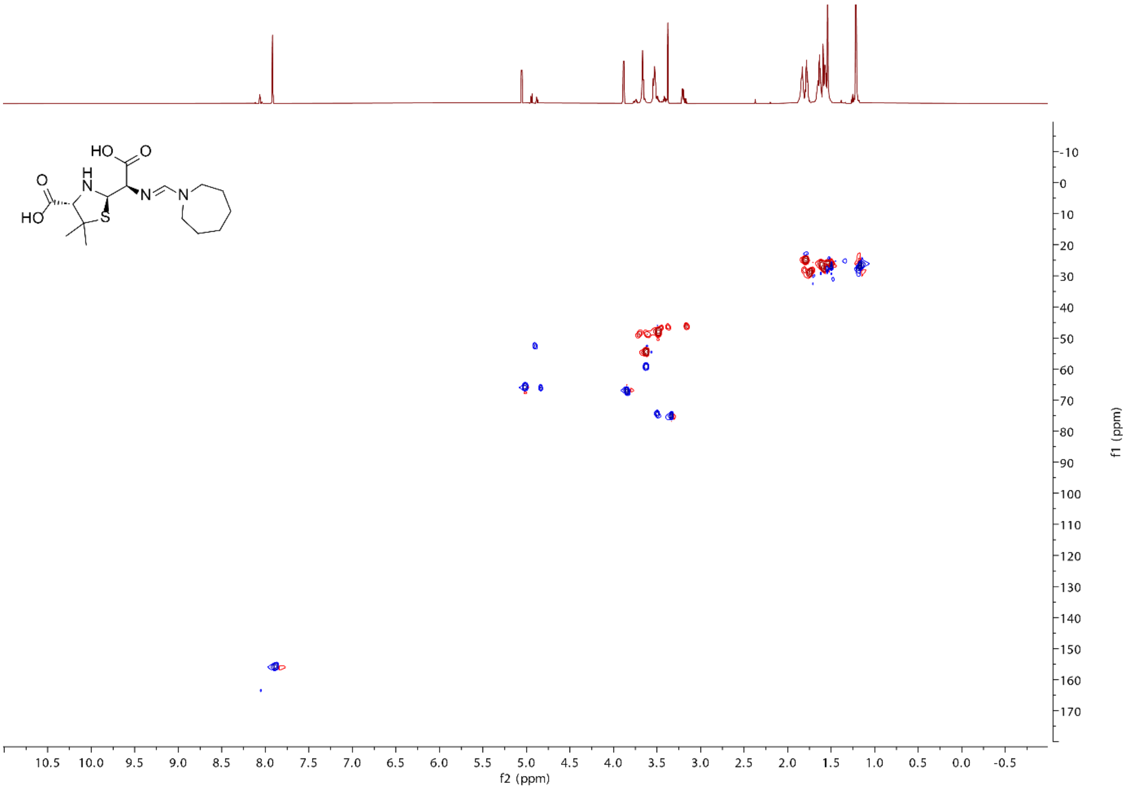

Figure S16. $^{1}H,^{13}C$ HSQC NMR spectrum (700 MHz, 25 mM Tris-d_{11} in D_2O) of hydrolysed mecillinam (**1**). Note, the 1H projection in the spectrum is the same as that shown in Figure S13 and is included here for easier visualisation.

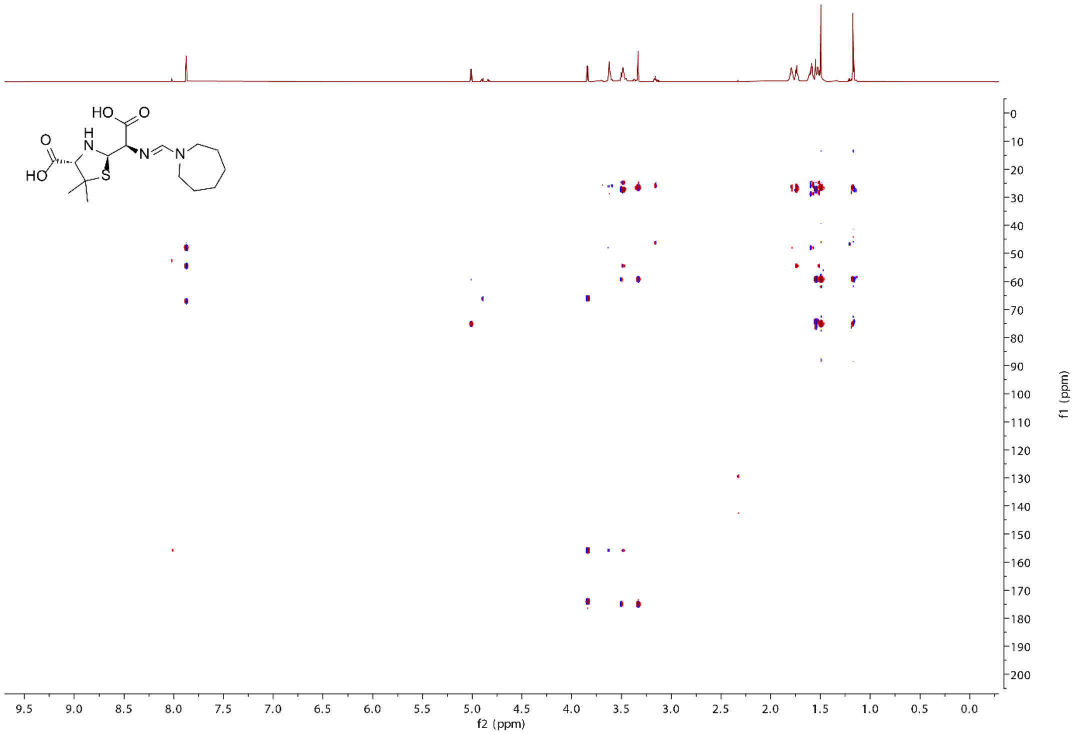

Figure S17. $^{1}H,^{13}C$ HMBC NMR spectrum (700 MHz, 25 mM Tris-d_{11} in D_2O) of hydrolysed mecillinam (**1**). Note, the 1H projection in the spectrum is the same as that shown in Figure S13 and is included here for easier visualisation.

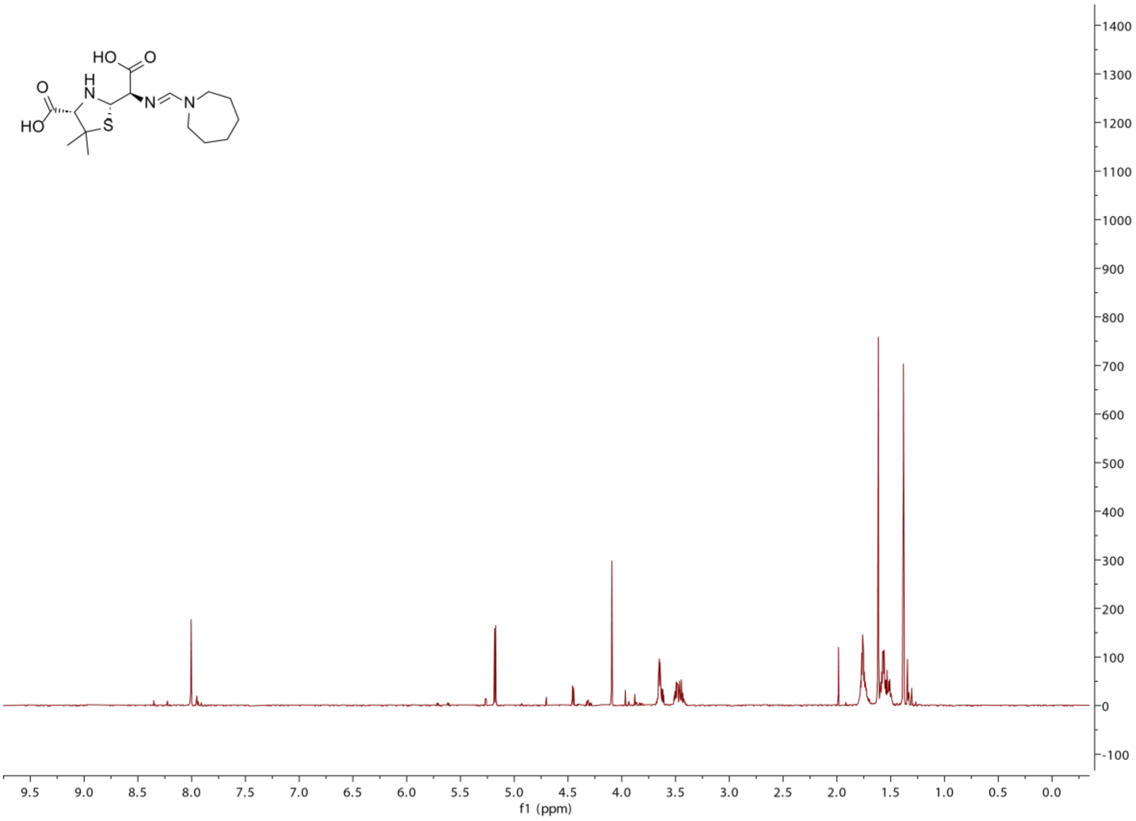

Figure S18. ^{1}H NMR spectrum (750 MHz, D_2O) of hydrolysed mecillinam epimer (**2**).

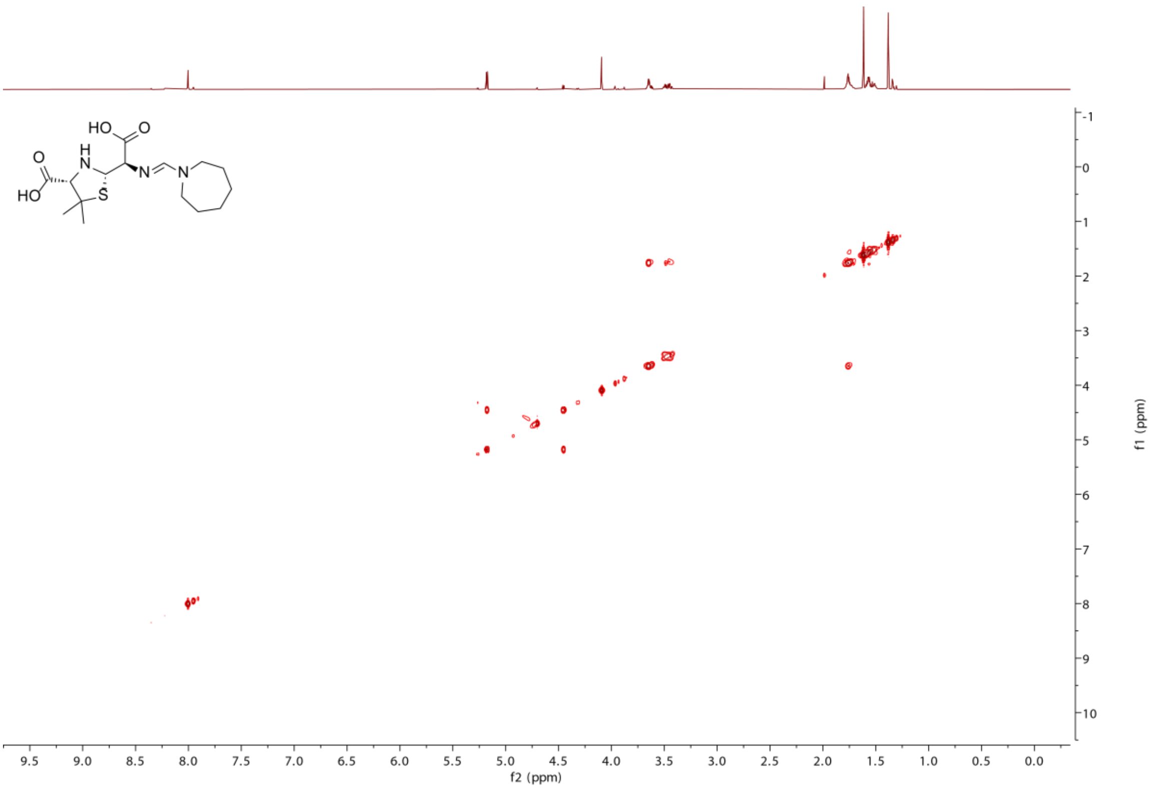

Figure S19. $^{1}H,^{1}H$ COSY NMR spectrum (750 MHz, D_2O) of hydrolysed mecillinam epimer (**2**). Note, the 1H projection in the spectrum is the same as that shown in Figure S18 and is included here for easier visualisation.

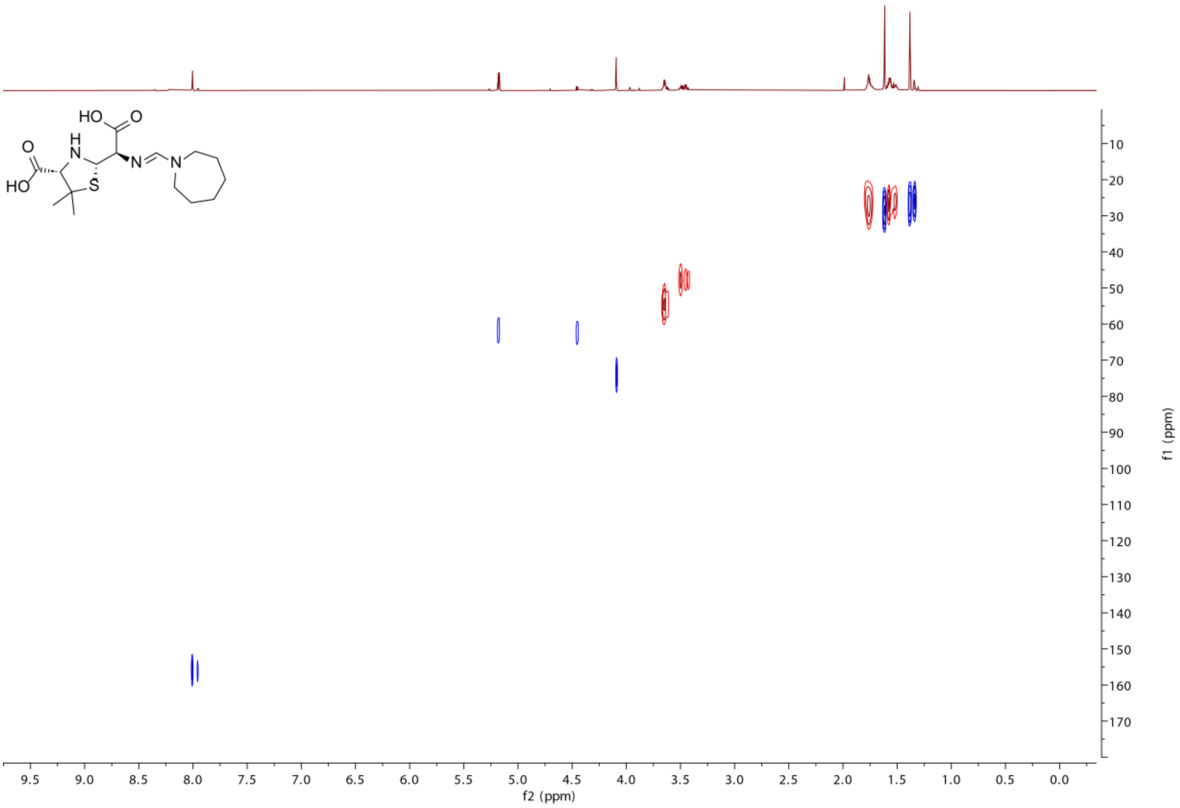

Figure S20. $^{1}H,^{13}C$ HSQC NMR spectrum (750 MHz, D_2O) of hydrolysed mecillinam epimer (**2**). Note, the 1H projection in the spectrum is the same as that shown in Figure S18 and is included here for easier visualisation.

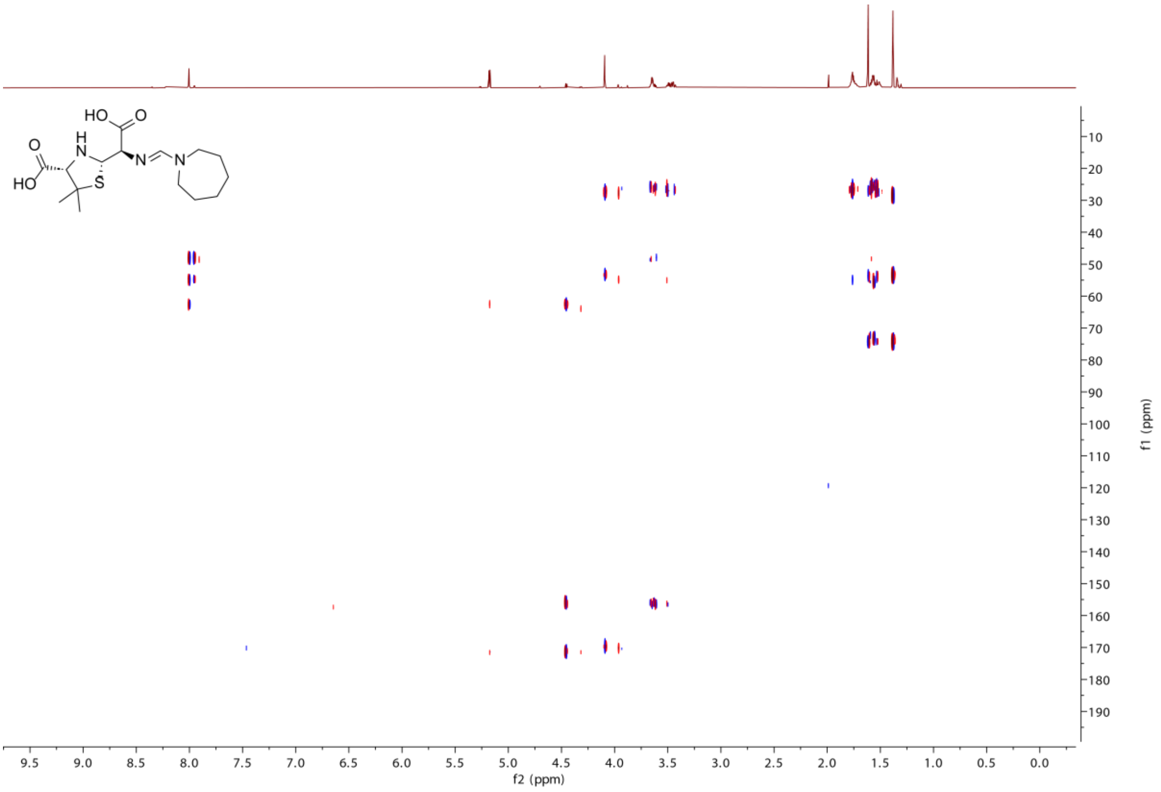

Figure S21. ^{1}H,^{13}C HMBC NMR spectrum (750 MHz, D_2O) of hydrolysed mecillinam epimer (**2**). Note, the 1H projection in the spectrum is the same as that shown in Figure S18 and is included here for easier visualisation.

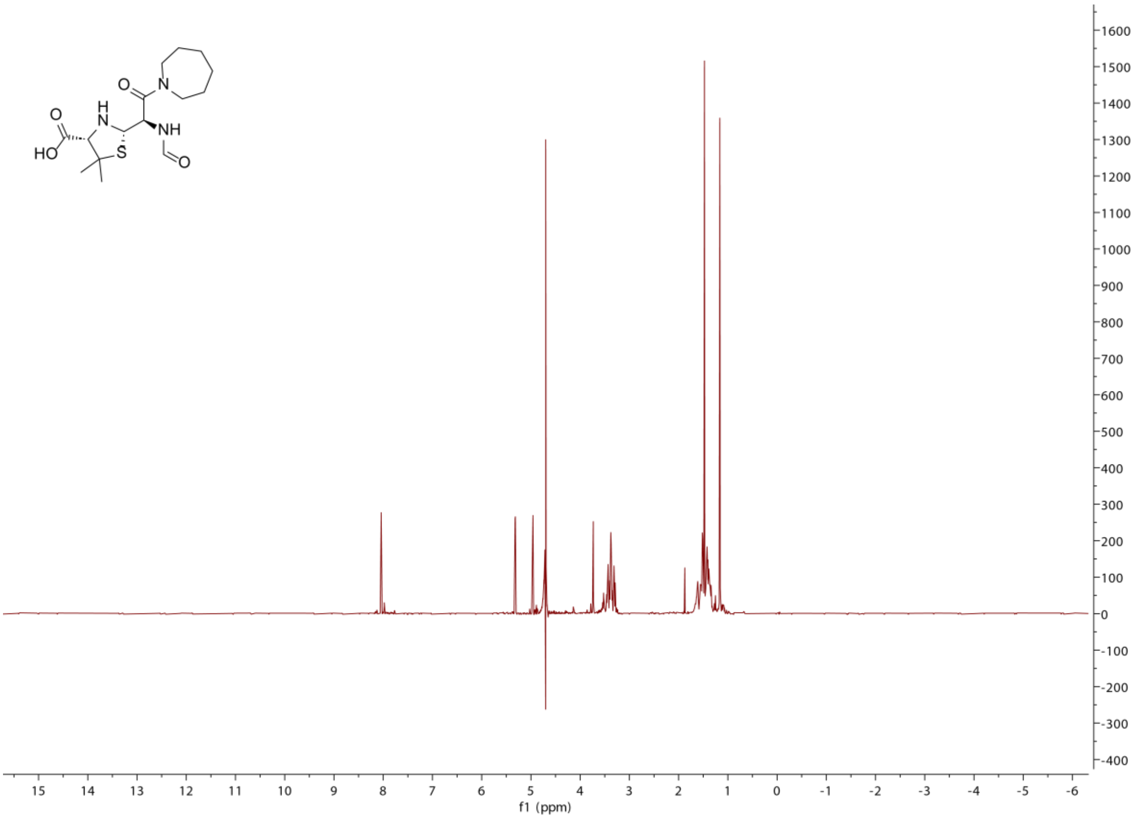

Figure S22. ^{1}H NMR spectrum (700 MHz, D_2O) of epimeric N-formyl amide (**3**).

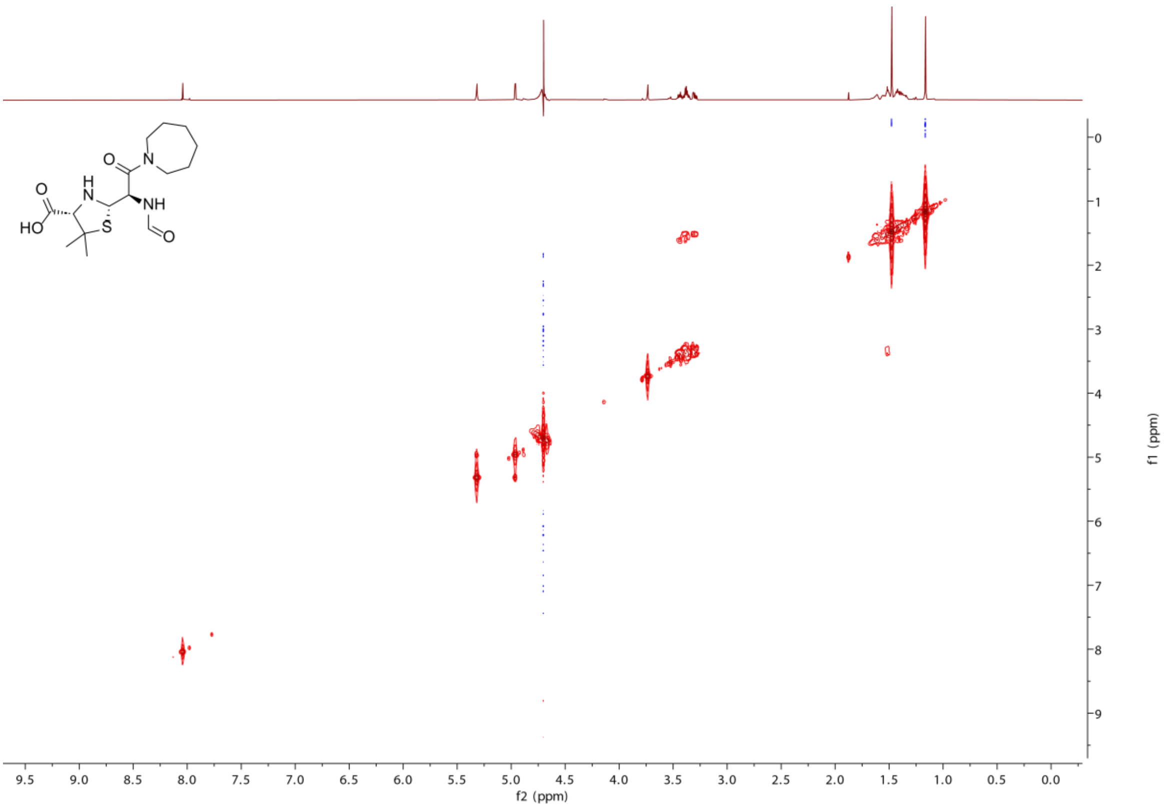

Figure S23. ^{1}H,^{1}H COSY NMR spectrum (700 MHz, D_2O) of epimeric N-formyl amide (**3**). Note, the 1H projection in the spectrum is the same as that shown in Figure S22 and is included here for easier visualisation.

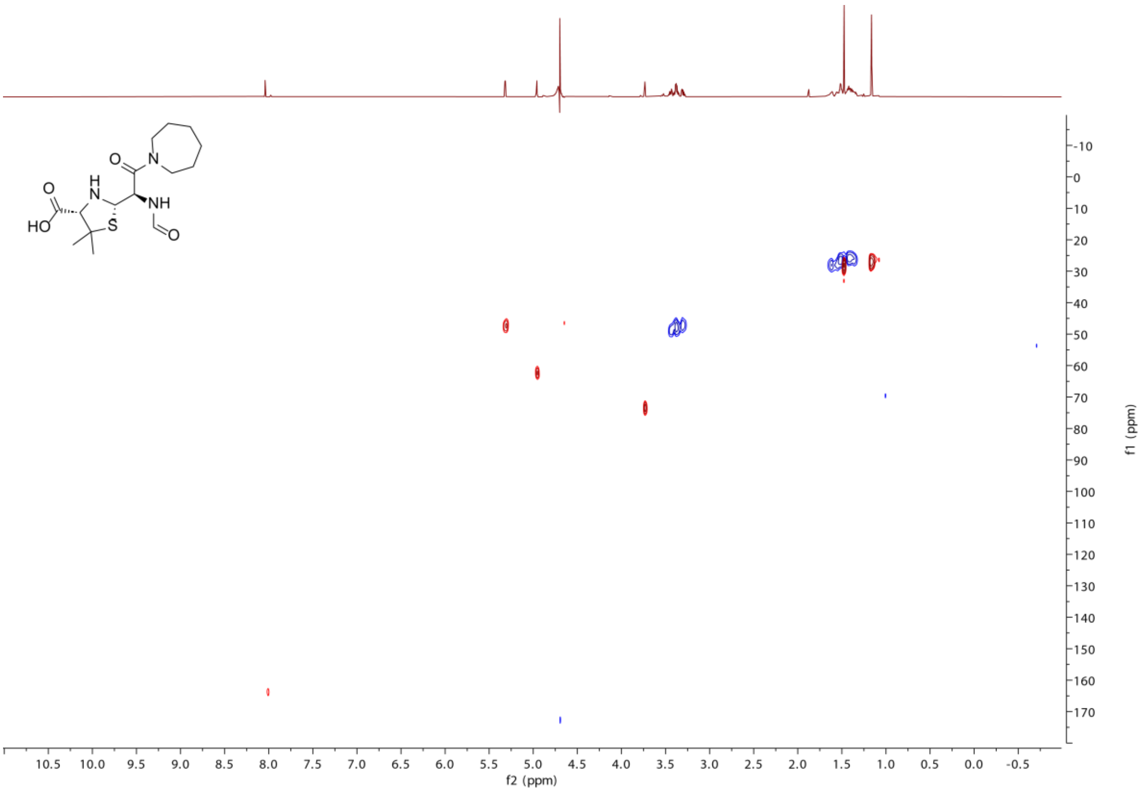

Figure S24. ^{1}H,^{13}C HSQC NMR spectrum (700 MHz, D_2O) of epimeric N-formylamide (**3**). Note, the 1H projection in the spectrum is the same as that shown in Figure S22 and is included here for easier visualisation.

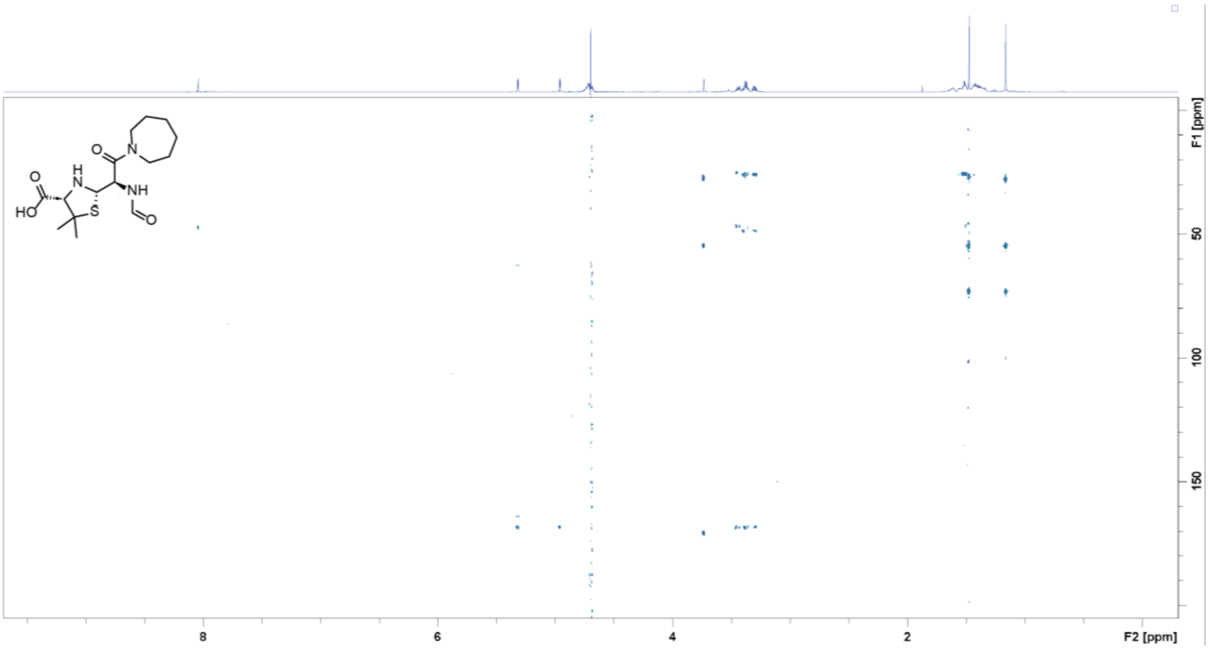

Figure S25. $^{1}H,^{13}C$ HMBC NMR spectrum (700 MHz, D_2O) of epimeric N-formyl amide (**3**). Note, the 1H projection in the spectrum is the same as that shown in Figure S22 and is included here for easier visualisation.

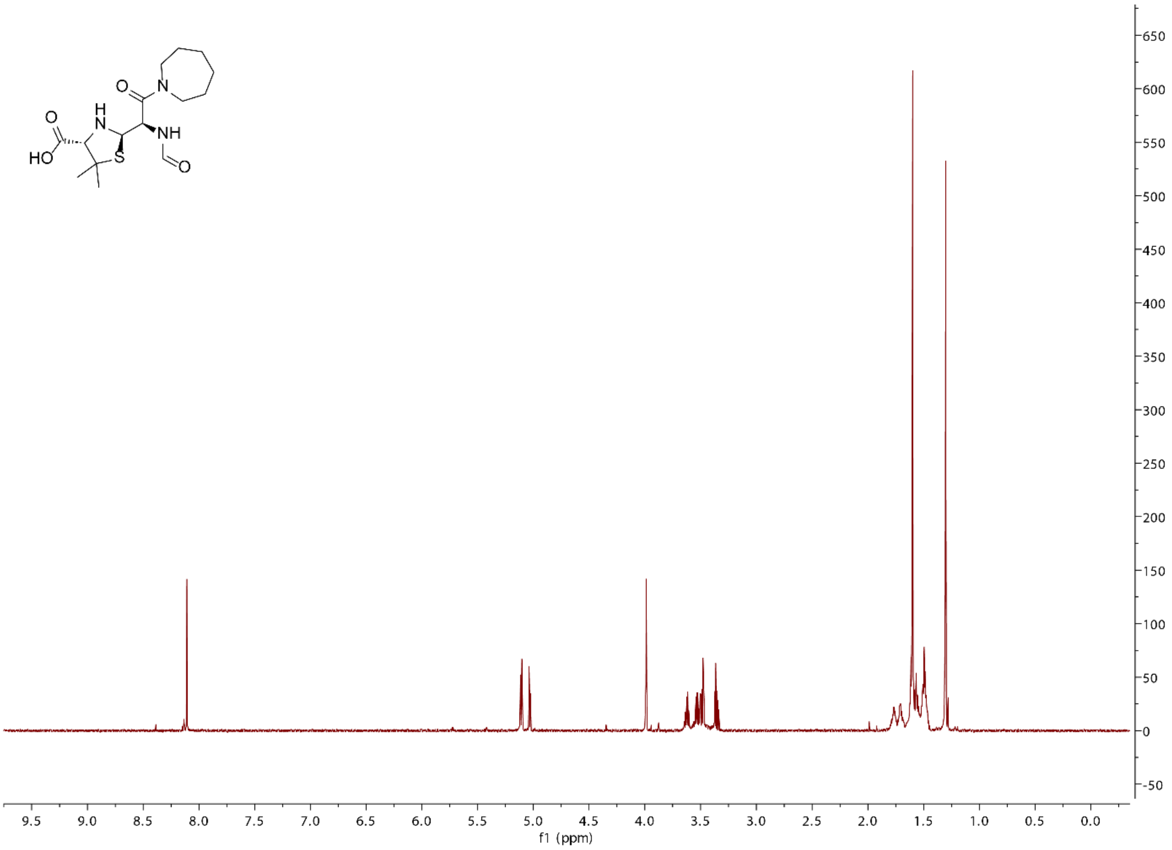

Figure S26. ^{1}H NMR spectrum (750 MHz, D_2O) of N-formyl amide (**4**).

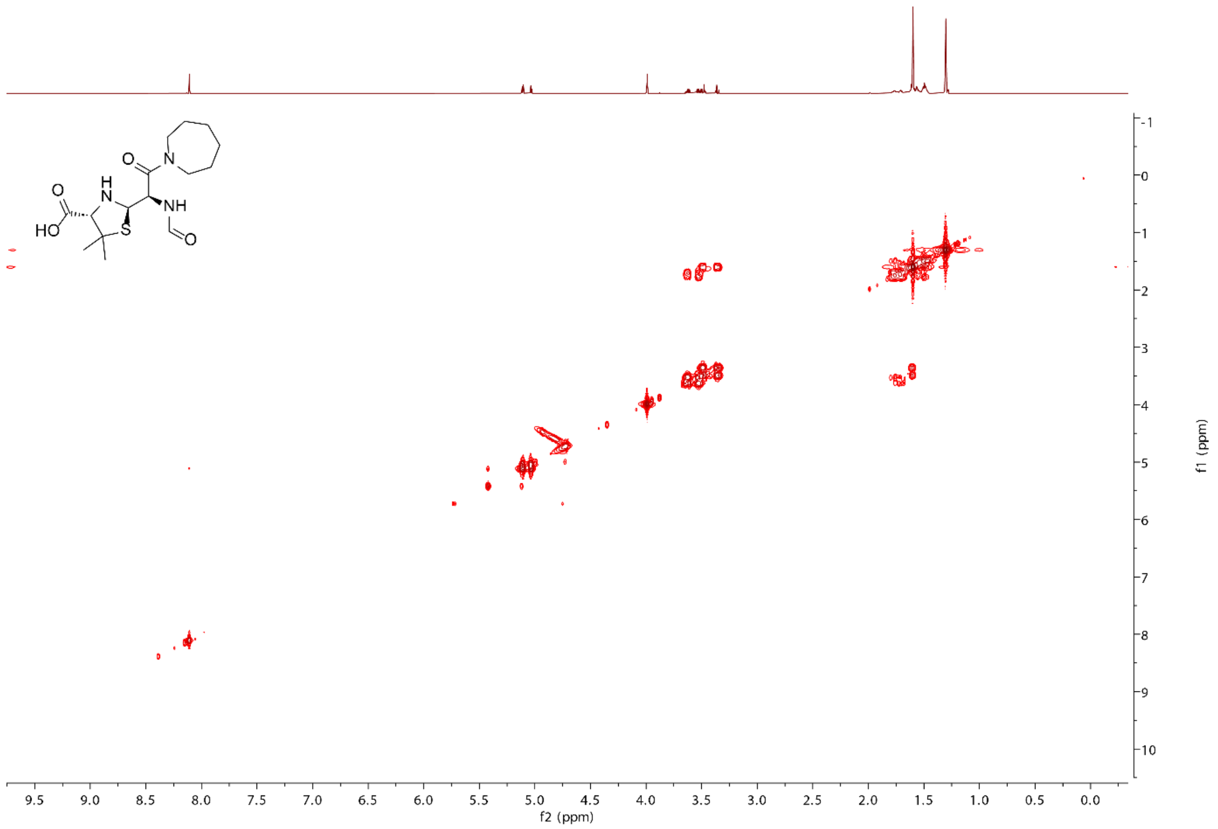

Figure S27. $^{1}H,^{1}H$ COSY NMR spectrum (750 MHz, D_2O) of N-formyl amide (**4**). Note, the 1H projection in the spectrum is the same as that shown in Figure S26 and is included here for easier visualisation.

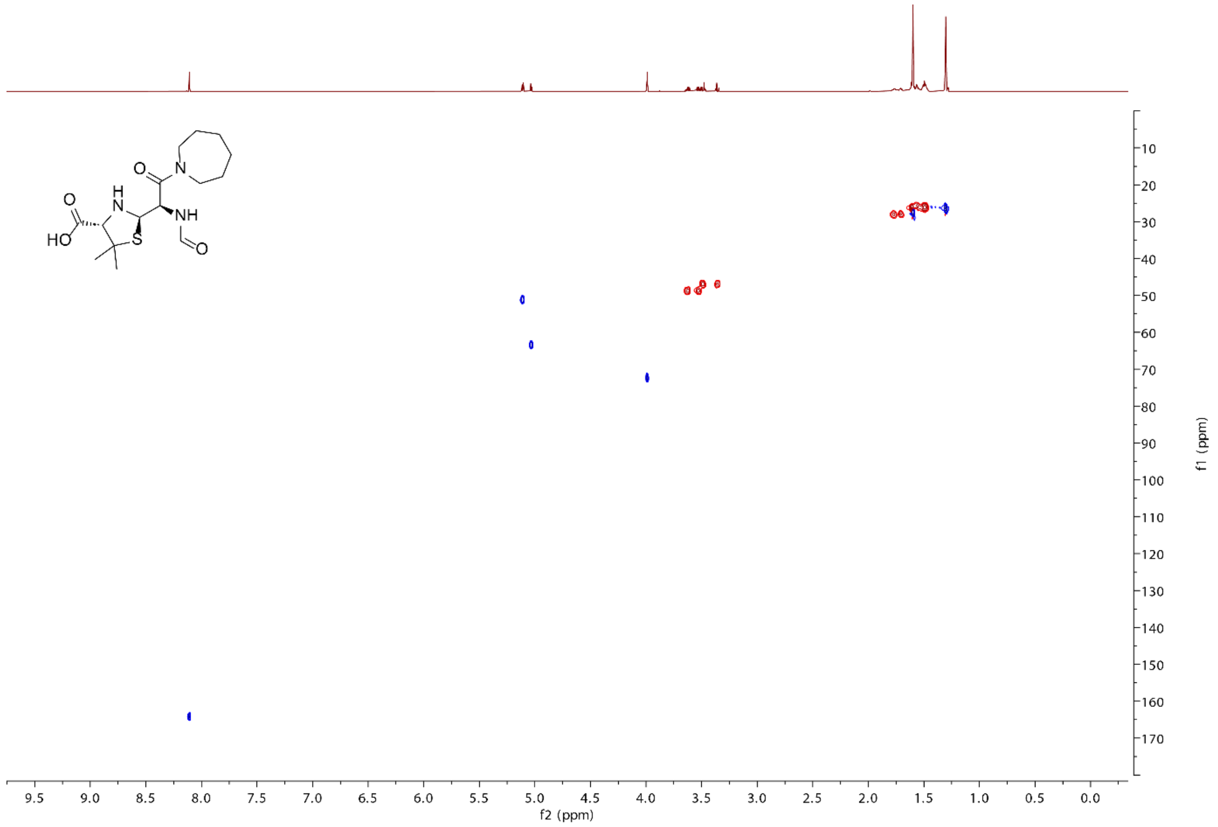

Figure S28. $^{1}H,^{13}C$ HSQC NMR spectrum (750 MHz, D_2O) of N-formyl amide (**4**). Note, the 1H projection in the spectrum is the same as that shown in Figure S26 and is included here for easier visualisation.

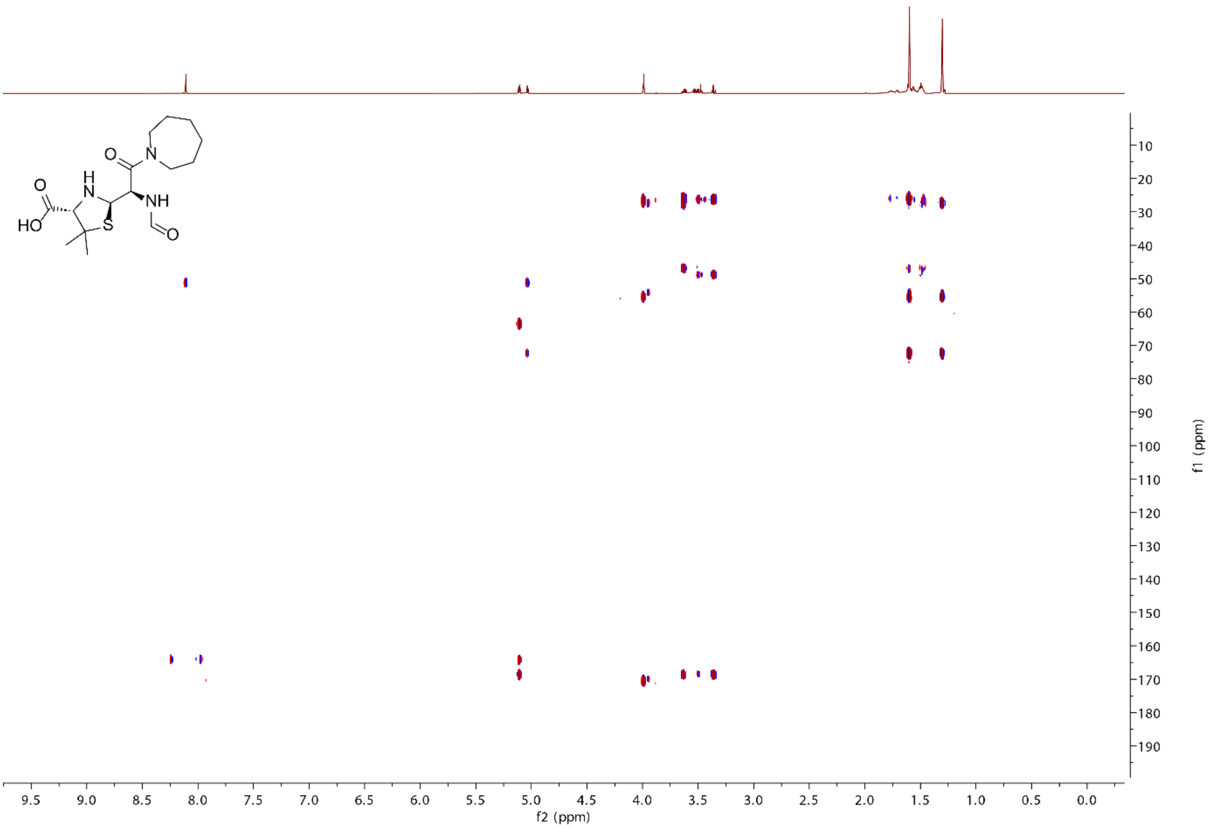

Figure S29. ^{1}H,^{13}C HMBC NMR spectrum (750 MHz, D_2O) of N-formyl amide (**4**). Note, the 1H projection in the spectrum is the same as that shown in Figure S26 and is included here for easier visualisation.

Supporting References

1. Wachino, J., Yamaguchi, Y., Mori, S., Jin, W., Kimura, K., Kurosaki, H., and Arakawa, Y. (2016) Structural Insights into Recognition of Hydrolyzed Carbapenems and Inhibitors by Subclass B3 Metallo-beta-Lactamase SMB-1. *Antimicrob Agents Chemother* **60**, 4274-4282
2. Baltzer, B., Lund, F., and Rastrup-Andersen, N. (1979) Degradation of mecillinam in aqueous solution. *J Pharm Sci* **68**, 1207-1215
3. Krajnc, A., Brem, J., Hinchliffe, P., Calvopina, K., Panduwawala, T. D., Lang, P. A., Kamps, J., Tyrrell, J. M., Widlake, E., Saward, B. G., Walsh, T. R., Spencer, J., and Schofield, C. J. (2019) Bicyclic Boronate VNRX-5133 Inhibits Metallo- and Serine-beta-Lactamases. *J Med Chem* **62**, 8544-8556
4. Brem, J., Panduwawala, T., Hansen, J. U., Hewitt, J., Liepins, E., Donets, P., Espina, L., Farley, A. J. M., Shubin, K., Campillos, G. G., Kiuru, P., Shishodia, S., Krahn, D., Lesniak, R. K., Schmidt Adrian, J., Calvopina, K., Turrientes, M. C., Kavanagh, M. E., Lubriks, D., Hinchliffe, P., Langley, G. W., Aboklaish, A. F., Eneroth, A., Backlund, M., Baran, A. G., Nielsen, E. I., Speake, M., Kuka, J., Robinson, J., Grinberga, S., Robinson, L., McDonough, M. A., Rydzik, A. M., Leissing, T. M., Jimenez-Castellanos, J. C., Avison, M. B., Da Silva Pinto, S., Pannifer, A. D., Martjuga, M., Widlake, E., Priede, M., Hopkins Navratilova, I., Gniadkowski, M., Belfrage, A. K., Brandt, P., Yli-Kauhaluoma, J., Bacque, E., Page, M. G. P., Bjorkling, F., Tyrrell, J. M., Spencer, J., Lang, P. A., Baranczewski, P., Canton, R., McElroy, S. P., Jones, P. S., Baquero, F., Suna, E., Morrison, A., Walsh, T. R., and Schofield, C. J. (2022) Imitation of beta-lactam binding enables broad-spectrum metallo-beta-lactamase inhibitors. *Nat Chem* **14**, 15-24

5. Hinchliffe, P., Tanner, C. A., Krismanich, A. P., Labbe, G., Goodfellow, V. J., Marrone, L., Desoky, A. Y., Calvopina, K., Whittle, E. E., Zeng, F., Avison, M. B., Bols, N. C., Siemann, S., Spencer, J., and Dmitrienko, G. I. (2018) Structural and Kinetic Studies of the Potent Inhibition of Metallo-beta-lactamases by 6-Phosphonomethylpyridine-2-carboxylates. *Biochemistry* **57**, 1880-1892
6. Abboud, M. I., Kosmopoulou, M., Krismanich, A. P., Johnson, J. W., Hinchliffe, P., Brem, J., Claridge, T. D. W., Spencer, J., Schofield, C. J., and Dmitrienko, G. I. (2018) Cyclobutanone Mimics of Intermediates in Metallo-beta-Lactamase Catalysis. *Chemistry* **24**, 5734-5737
7. Ullah, J. H., Walsh, T. R., Taylor, I. A., Emery, D. C., Verma, C. S., Gamblin, S. J., and Spencer, J. (1998) The crystal structure of the L1 metallo-beta-lactamase from Stenotrophomonas maltophilia at 1.7 A resolution. *J Mol Biol* **284**, 125-136
8. Raczynska, J. E., Shabalin, I. G., Minor, W., Wlodawer, A., and Jaskolski, M. (2018) A close look onto structural models and primary ligands of metallo-beta-lactamases. *Drug Resist Updat* **40**, 1-12
9. Feng, H., Ding, J., Zhu, D., Liu, X., Xu, X., Zhang, Y., Zang, S., Wang, D. C., and Liu, W. (2014) Structural and mechanistic insights into NDM-1 catalyzed hydrolysis of cephalosporins. *J Am Chem Soc* **136**, 14694-14697
10. Spencer, J., Read, J., Sessions, R. B., Howell, S., Blackburn, G. M., and Gamblin, S. J. (2005) Antibiotic recognition by binuclear metallo-beta-lactamases revealed by X-ray crystallography. *J Am Chem Soc* **127**, 14439-14444
